# Characterization of molecular mechanisms driving Merkel cell polyomavirus oncogene transcription and tumorigenic potential

**DOI:** 10.1371/journal.ppat.1011598

**Published:** 2023-08-30

**Authors:** June F. Yang, Wei Liu, Jianxin You

**Affiliations:** Department of Microbiology, Perelman School of Medicine, University of Pennsylvania, Philadelphia, Pennsylvania, United States of America; Tufts University School of Medicine, UNITED STATES

## Abstract

Merkel cell polyomavirus (MCPyV) is associated with approximately 80% of cases of Merkel cell carcinoma (MCC), an aggressive type of skin cancer. The incidence of MCC has tripled over the past twenty years, but there are currently very few effective targeted treatments. A better understanding of the MCPyV life cycle and its oncogenic mechanisms is needed to unveil novel strategies for the prevention and treatment of MCC. MCPyV infection and oncogenesis are reliant on the expression of the early viral oncoproteins, which drive the viral life cycle and MCPyV+ MCC tumor cell growth. To date, the molecular mechanisms regulating the transcription of the MCPyV oncogenes remain largely uncharacterized. In this study, we investigated how MCPyV early transcription is regulated to support viral infection and MCC tumorigenesis. Our studies established the roles of multiple cellular factors in the control of MCPyV gene expression. Inhibitor screening experiments revealed that the histone acetyltransferases p300 and CBP positively regulate MCPyV transcription. Their regulation of viral gene expression occurs through coactivation of the transcription factor NF-κB, which binds to the viral genome to drive MCPyV oncogene expression in a manner that is tightly controlled through a negative feedback loop. Furthermore, we discovered that small molecule inhibitors specifically targeting p300/CBP histone acetyltransferase activity are effective at blocking MCPyV tumor antigen expression and MCPyV+ MCC cell proliferation. Together, our work establishes key cellular factors regulating MCPyV transcription, providing the basis for understanding the largely unknown mechanisms governing MCPyV transcription that defines its infectious host cell tropism, viral life cycle, and oncogenic potential. Our studies also identify a novel therapeutic strategy against MCPyV+ MCC through specific blockage of MCPyV oncogene expression and MCC tumor growth.

## Author summary

MCPyV is a ubiquitous skin infection that can cause one of the most aggressive and highly fatal skin cancers, MCC, which has been increasing in incidence in the decades since its initial discovery. Despite the growing concern presented by this cancer, there are currently no effective targeted therapies to treat MCC. MCC metastasizes rapidly and resists currently available chemotherapy and immune checkpoint inhibitor treatment strategies in a significant portion of patients. Approximately 80% of MCCs are caused by MCPyV, which normally maintains asymptomatic infection within the skin but in rare cases drives MCPyV+ MCC oncogenesis through the expression of the viral oncogenes. Our characterization of the largely unknown molecular mechanisms controlling MCPyV gene expression furthers our understanding of the link between MCPyV infection and MCC oncogenesis. Our study also identifies druggable targets that are exploitable to specifically repress MCPyV oncogene expression and MCC tumor growth. This work demonstrates that hampering viral oncogene transcription is a novel and effective therapeutic strategy to obliterate MCPyV-induced MCC tumorigenesis.

## Introduction

Merkel cell carcinoma (MCC) is an aggressive type of skin cancer initially described in 1972 [1,2]. Though rare, the incidence of MCC has more than tripled in the decades since its discovery [3–5]. MCC is a fatal cancer, with a 3-year mortality rate (33%) exceeding that of melanoma (15%) [6]. Metastatic disease is associated with exceptionally poor prognoses [6], and is not reliably treatable with currently available strategies [7–10]. MCC is resistant to chemotherapy and progresses even in patients that respond to treatment [10]. Though PD-1/PD-L1 inhibitors have shown more durable responses in clinical trials than traditional chemotherapeutics, approximately 50% of patients do not respond to this therapy [7–9]. The abilities of MCC to metastasize rapidly and to resist currently available therapies reveal an ongoing need for the development of novel and targeted treatments against this highly aggressive cancer.

Merkel cell polyomavirus (MCPyV) was discovered in 2008 as the etiological agent associated with approximately 80% of MCC cases [11,12]. Normally, MCPyV is associated with widespread asymptomatic infection that persists primarily within the skin [13–15]. During persistent infection, MCPyV is maintained as an episomal double-stranded DNA virus with a 5.4 kb genome [16,17]. The viral genome encodes the expression of several products, regulated by a single noncoding regulatory region (NCRR) which contains promoters driving the bidirectional and temporally regulated expression of viral genes during infection [16,18,19]. The MCPyV early promoter (EP) drives the expression of the large and small tumor antigens (LT and sT), as well as the 57kT antigen and the Alternative Large T Open reading frame (ALTO) [11,16,20]. Of these viral early gene products, LT and sT are the best characterized, and have been shown to fulfill several functions within the MCPyV life cycle, including the initiation of viral replication [18,19,21–23]. The MCPyV late promoter (LP) drives the expression of VP1 and VP2, which assemble to form the viral capsid [24,25], and an miRNA [26]. Though MCPyV has been shown to promiscuously enter multiple cell types, viral gene expression only occurs in a highly restricted host cell range that includes only MCPyV+ MCC cells and human dermal fibroblasts (HDFs) [25,27–30]. Our group discovered that, within the human skin, MCPyV virions are capable of entering both HDFs and human foreskin keratinocytes (HFKs), but viral gene expression is detected only in HDFs [28,30,31]. The MCPyV promoters are also silenced in many other nonpermissive cell types [27,28,30]. Together, the relative promiscuity of MCPyV entry and the highly restricted host cell range supporting MCPyV gene expression indicate that cell type-specific epigenetic modifications and/or regulatory factors drive the highly constricted MCPyV transcriptional activity that defines the virus’ narrow host cell tropism and, as discussed below, its oncogenic potential.

In MCPyV+ MCCs, the MCPyV DNA is no longer episomal, and is instead clonally integrated within the genome of the tumor cells [11,12]. The integrated viral genome in MCPyV+ MCC invariably contains an intact NCRR [32,33], in which the MCPyV EP activates the constitutive expression of sT and a truncated form of LT (LTT) which retains its retinoblastoma protein (Rb)-binding LXCXE motif but lacks replicative function due to disruptions of its helicase domain [11,29,33]. In these tumors, sT and LTT function as the key viral oncogenes to promote MCC tumor growth [11,23,29,32–43]. It has become clear that MCPyV+ MCCs are addicted to the continued expression of sT/LTT from the integrated viral genome and do not survive inhibition of tumor oncogene expression [16,37,39].

The MCPyV EP therefore plays a critical role in both the infectious life cycle of MCPyV and the oncogenic progression of MCPyV+ MCC cells. Understanding the mechanisms controlling the transcription of the MCPyV tumor antigens would therefore shed light on how MCPyV establishes infection within its highly narrow host cell range [28,30], and how dysregulation of viral oncogene expression may contribute to MCC tumorigenesis [37,40,41]. In addition, because of the important role of MCPyV EP-mediated transcription in driving MCC tumor growth [11,23,29,32,33,35–42], elucidation of the molecular mechanisms underlying EP-regulated oncogene expression in MCPyV+ MCC could identify molecular targets that can be exploited to inhibit tumor antigen expression in cancer cells, offering a targeted treatment strategy against this aggressive cancer [37,39]. However, though the regulatory mechanisms controlling the transcription of closely related viruses such as simian virus 40 (SV40), JC polyomavirus (JCPyV), and BK polyomavirus (BKPyV) have been well-characterized [44,45], very little is currently known about how MCPyV EP transcription is regulated during either viral infection or MCC development [42,44,46,47].

In this study, we investigated the epigenetic modification enzymes and transcription factors that regulate MCPyV early transcription. We discovered that the closely related histone acetyltransferases, p300 and CREB-binding protein (CBP), play a critical role in MCPyV early transcription through coactivation of the p65 subunit of the transcription factor NF-κB. We found that NF-κB function is important to support MCPyV EP transcription. However, over-stimulated p65 activity represses viral gene expression through a negative feedback loop, supporting a tightly regulated MCPyV transcriptional control mechanism that controls viral latency. Additionally, we found that blocking p300/CBP activity with small molecule inhibitors led to the specific killing of MCPyV+ MCC cells via inhibition of viral oncoprotein expression. Our studies therefore reveal a new potential therapeutic strategy for treating MCPyV+ MCC.

## Results

### MCPyV EP is specifically activated in MCPyV+ MCC cells and HDFs, but not keratinocytes

**Fig 1:**
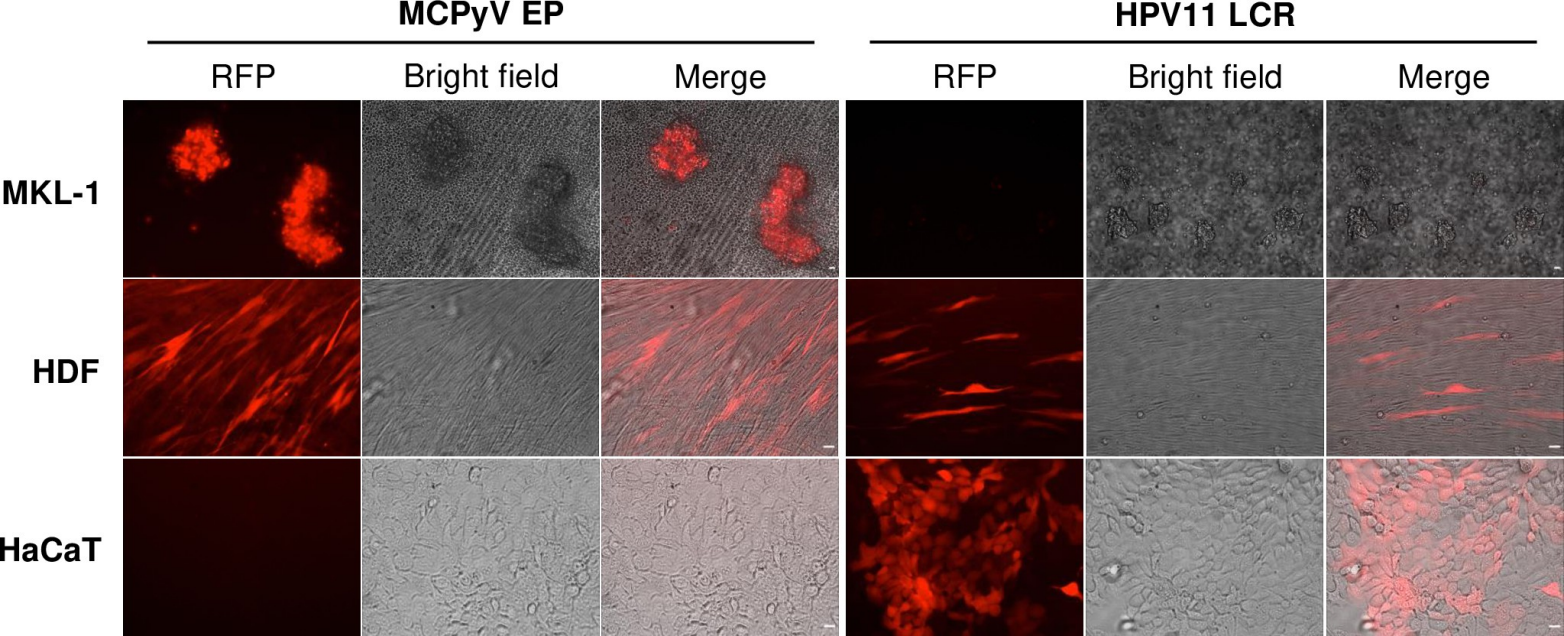
MCPyV EP is specifically activated in the MCPyV+ MCC cell line MKL-1 and normal HDFs, but not in keratinocytes. Lentiviruses carrying MCPyV EP-RFP or HPV11 LCR-RFP were used to infect MKL-1, HDFs and the keratinocyte cell line HaCaT. The stable cells were imaged using an inverted fluorescence microscope (IX81; Olympus). HPV11 LCR-RFP preferentially expressed in keratinocytes serves as a control for keratinocyte viability. Bar: 20μm.

Studies on the polyomaviruses SV40, JCPyV, and BKPyV indicate that the NCRR contains promoter and enhancer elements that are regulated by both epigenetic modifications and transcription factors to drive the expression of the viral genes [44]. To test whether the MCPyV NCRR also contains elements regulating viral transcription, we stably transduced the MCPyV+ MCC cell line MKL-1, MCPyV-permissive HDFs, or nonpermissive keratinocytes with lentivirus encoding an RFP reporter under the control of either the MCPyV EP or a control DNA element, the HPV11 LCR (Fig 1). MCPyV EP-driven RFP reporter expression was only detected in MKL-1 and HDFs, and not in keratinocytes (Fig 1). Keratinocytes supported the robust expression of the HPV11 LCR-driven RFP reporter, indicating that the lack of MCPyV EP-RFP expression in keratinocytes was not due to failed lentiviral transduction (Fig 1). This result confirmed that the integrated EP-reporter construct was able to recapitulate MCPyV EP transcription in MCPyV+ MCC cells, while being completely silent in keratinocytes (Fig 1). Our findings corroborated previous studies in which it was found that, while MCPyV can promiscuously enter many types of cells in the skin, viral early gene expression is supported in only a few cell types such as HDFs and MCPyV+ MCC cells, but silenced in nonpermissive cells such as keratinocytes [28,30,31,46]. From this finding, we conclude that the MCPyV EP contains the elements through which the cell-type specific gene expression of this virus is regulated. Cells carrying MCPyV EP-reporters therefore provide an excellent platform for identifying host regulatory factors essential for supporting EP transcriptional activity.

### Exploring the epigenetic mechanisms regulating MCPyV NCRR-driven transcription

Epigenetic modifications play an important role in transcriptional regulation [48]. Histone H3 and H4 acetylation and H3K4/H3K36 methylation are associated with transcriptional activation, whereas tri-methylation of H3K9 and H3K27 is linked to transcriptional repression. In addition, DNA methylation is associated with transcriptional silencing [49,50]. Previously, transactivating histone marks were detected in the NCRR of MCPyV genomes transfected into PFSK-1 cells that support MCPyV gene expression [51]. This finding suggests that, similar to those of closely related polyomaviruses [44], the MCPyV NCRR is likely regulated by histone modifications. SDS-PAGE and SYPRO Ruby protein staining of purified MCPyV virions have detected encapsidated histones [24], further confirming that MCPyV DNA is packaged into histone-bound nucleosomes likely carrying epigenetic modifications that can control its transcription in infected cells. To determine the impact of epigenetic modifications on MCPyV transcription, we conducted a screening to assess the effects of several epigenetic enzyme inhibitors on LT expression in cells transfected with full MCPyV genomes (S1 Fig). Treatment of MCPyV-transfected cells with inhibitors against histone acetyltransferases (HATs), histone deacetylases (HDACs), and bromodomain and extra-terminal (BET) proteins repressed the expression of MCPyV LT in transfected cells (S1 Fig). In contrast, inhibitors against DNA methyltransferases or histone-lysine methyltransferases did not affect MCPyV LT expression (S1 Fig). Therefore, besides BET inhibitors, which prevent the binding of BET proteins to acetylated histones or factors [52], only inhibitors that affect histone acetylation downregulated LT expression in MCPyV-transfected cells (S1B Fig). Furthermore, several of the effective HAT inhibitors (HATis) used in this screen specifically inhibit the closely related HATs p300 and CBP (S1A Fig). We therefore further investigated the effects of p300/CBP-mediated acetylation on MCPyV EP-mediated transcription.

### MCPyV EP-driven transcription is regulated by p300/CBP acetyltransferase activity

**Fig 2:**
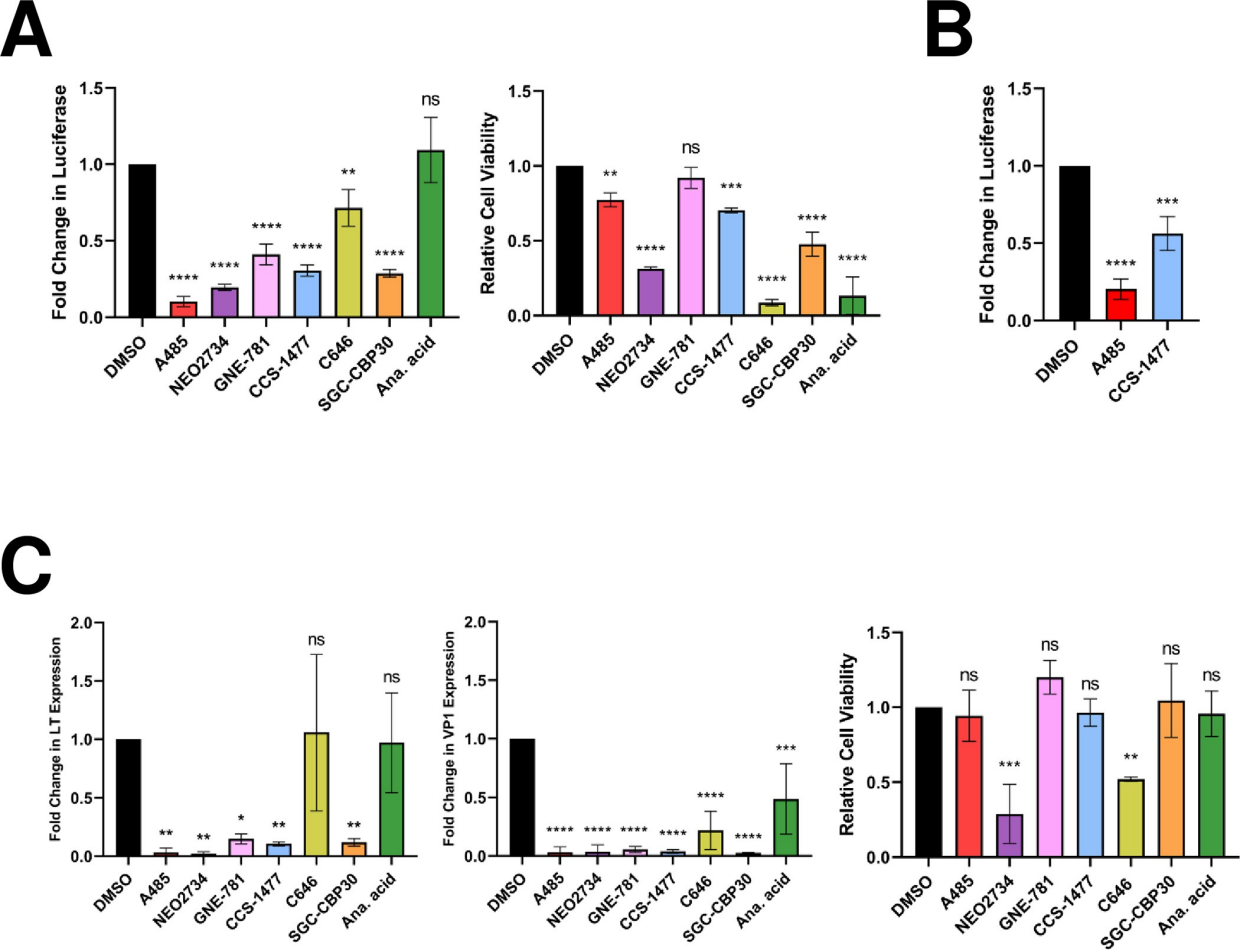
HATi treatment represses MCPyV EP-driven transcription. (A) HEK293 cells stably expressing an MCPyV EP-luciferase reporter were treated with DMSO, 2 μM A485, 1 μM NEO2734, 1 μM GNE-781, 1 μM CCS-1477, 10 μM C646, 10 μM SGC-CBP30, or 20 μM anacardic acid for 72h, then collected for luciferase or CellTiterGlo 3D assays. (B) HDFs stably expressing an MCPyV EP-luciferase reporter were treated with DMSO, 250 nM A485, or 250 nM CCS-1477 for 72h, then collected for luciferase assays. For both (A) and (B), fold changes in Luciferase were calculated after luciferase readings were normalized to the total protein concentration of each sample. (C) MCPyV-infected HDFs were treated with DMSO, 2 μM A485, 1 μM NEO2734, 1 μM GNE-781, 1 μM CCS-1477, 10 μM C646, 10 μM SGC-CBP30, or 20 μM anacardic acid on day 2 post-infection. Cells were collected on day 5 post-infection for CellTiterGlo 3D assays or RT-qPCR analysis of viral mRNA. RT-qPCR quantifications of viral mRNA expression were normalized to levels of cellular GAPDH mRNA. Error bars represent the standard deviation of three independent experiments. ****p<0.0001; ***p<0.001; **p<0.01; *p<0.05; ns = not significant.

To verify the results of the initial epigenetic inhibitor study, we examined the effect of an extended panel of HATis (A485, NEO2734, GNE-781, CCS-1477, C646, SGC-CBP30, and anacardic acid) on MCPyV EP activity. Though these compounds all inhibit p300/CBP [53–59], NEO2734 and anacardic acid have additional molecular targets [55,60], while C646 and anacardic acid also have other nonspecific effects [61]. Nonetheless, this varied group of inhibitors was selected to establish a general pattern of the impact of p300/CBP-specific activity on viral transcription. With the exception of anacardic acid, these inhibitors could effectively repress p300/CBP-specific histone 3 lysine 27 acetylation (H3K27ac) in HDFs, demonstrating their activity against p300/CBP (S2 Fig). Treatment with these HATis repressed the expression of an MCPyV EP-luciferase reporter stably maintained in both HEK293 and HDFs (Fig 2A and 2B), demonstrating that acetylation positively regulates MCPyV EP-driven gene expression. Treatment with these HATis also significantly downregulated both LT and VP1 mRNA expression in HDFs infected with native MCPyV virions (Fig 2C), further supporting that acetylation by p300/CBP regulates viral transcription during the MCPyV infectious life cycle. Notably, the compounds NEO2734, C646, and anacardic acid were generally toxic to treated cells (Fig 2A and 2C), as expected by their relatively nonspecific targeting of p300/CBP [55,61]. However, toxicity alone did not account for a decrease in MCPyV EP activity (Fig 2A and 2C, compare between luciferase readings and cell viability for C646 and anacardic acid). By normalizing the luciferase or MCPyV gene expression levels to the total protein concentration in each sample, we were able to account for changes in cell viability. Together, our data suggest that specific targeting of the EP contributes to the downregulation of reporter and LT expression.

**Fig 3:**
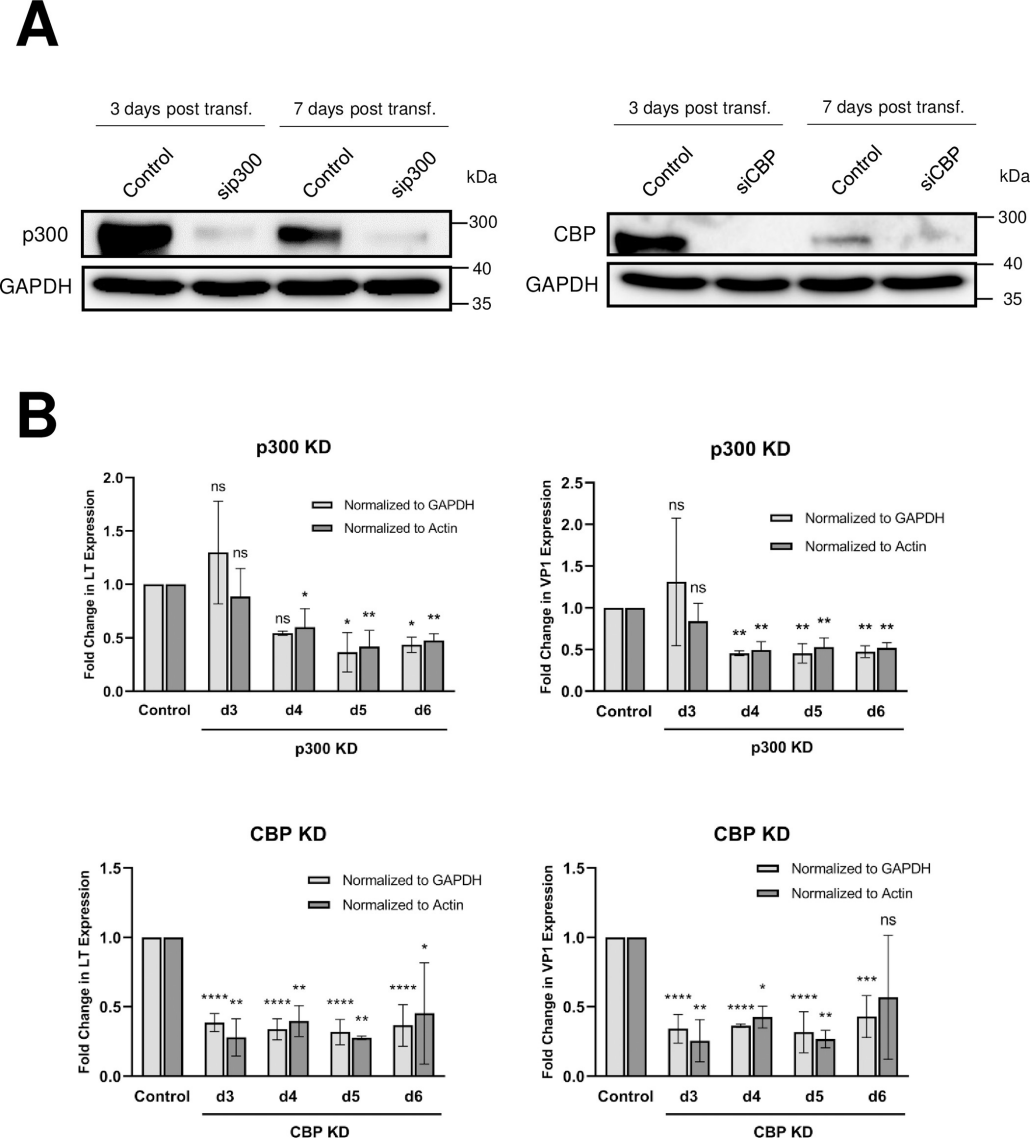
p300 and CBP are important for supporting MCPyV transcription during infection. (A) Whole cell lysates of HDFs transfected with siRNA targeting p300 (sip300), CBP (siCBP), or a scrambled control were collected at d3 and d7 post-transfection for Western blot analysis. (B) HDFs were transfected with siRNA against p300 (sip300), CBP (siCBP), or a scrambled control 24h prior to infection with 10^8^ viral genome equivalents of MCPyV. RT-qPCR analysis of viral mRNA expression in MCPyV-infected HDFs was performed on days 3 through 6 post-infection. Changes in LT and VP1 expression in the KD cells relative to the levels of viral transcription in control siRNA-transfected HDFs were calculated and normalized to cellular levels of GAPDH or actin mRNA as indicated. Error bars represent the standard deviation of three independent experiments. ****p<0.0001; ***p<0.001; **p<0.01; *p<0.05; ns = not significant.

**Fig 4:**
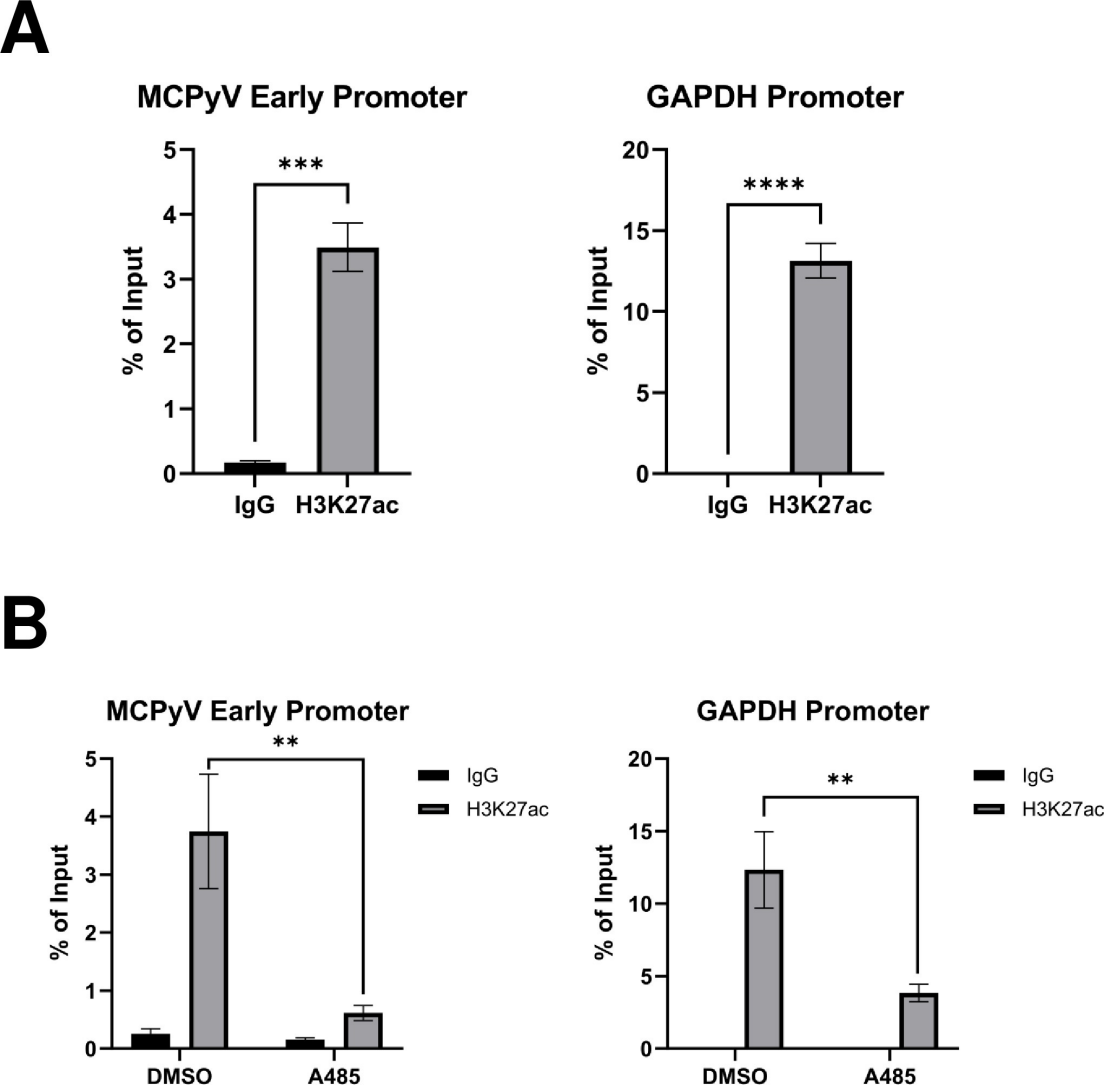
Detection of p300/CBP-specific histone acetylation marks on the MCPyV EP. ChIP was performed with MKL-1 cells (A) or MKL-1 cells that have been treated for 1h with DMSO or 2 uM A485 (B) using 0.5 μg normal rabbit IgG or antibody recognizing the p300/CBP-specific histone acetylation mark H3K27ac. qPCR was performed on the ChIP samples using primers recognizing the MCPyV EP or the GAPDH promoter. Error bars represent the standard deviation of three independent experiments. ****p<0.0001; ***p<0.001; **p<0.01.

We then adopted a genetic approach to confirm that the inhibitors repressed MCPyV gene expression through their specific suppression of p300/CBP activity, and not through off-target effects associated with drug treatment. We performed siRNA-mediated knockdown of p300 and CBP in HDFs prior to MCPyV infection (Fig 3A). Knockdown of either p300 or CBP significantly reduced LT and VP1 mRNA levels in MCPyV-infected HDFs (Fig 3B), confirming that p300/CBP plays an important role in the regulation of viral transcription. Through chromatin immunoprecipitation (ChIP) assays, we also detected the p300/CBP-specific histone acetylation mark H3K27ac on the MCPyV EP in the MCPyV+ MCC cell line MKL-1 (Fig 4A). Furthermore, treatment of MKL-1 cells with the HATi A485 significantly reduces EP-associated H3K27ac (Fig 4B), demonstrating that p300/CBP directly acetylate histones associated with the viral transcription control region.

To further examine the positive effect of HAT activity on MCPyV EP-driven gene expression, we used HDACi treatment to preserve acetylation in HEK293 cells expressing an MCPyV EP-luciferase reporter. Indeed, reporter expression was significantly upregulated in cells treated with low concentrations of HDACi (S3 Fig). Together, our findings support that acetyltransferase activity of the HATs p300/CBP positively regulates MCPyV gene expression.

### NF-κB regulates MCPyV gene expression in a tightly regulated manner

In addition to histone modification by epigenetic enzymes, sequence-specific binding of transcription factors to regulatory elements within the genome is another major mode of transcriptional regulation. *In silico* analyses have predicted that several well-characterized transcription factors, including NF-κB, bind to the MCPyV NCRR [62]. Our group recently discovered that the p65 subunit of NF-κB is activated during MCPyV infection and localized within the nuclei of MCPyV-infected LT-expressing HDFs [63]. Furthermore, p300/CBP are known to modulate the transcription of cellular genes by acting as coactivators of DNA-binding transcription factors [64]. Among the factors coactivated by p300/CBP is NF-κB, which is acetylated on multiple residues of its p65 subunit after its translocation to the nucleus in activated cells [65,66]. Taken together, the presence of NF-κB p65 in the nuclei of MCPyV infected cells actively expressing MCPyV LT [63], NF-κB’s predicted sequence-specific binding of the NCRR [62], and the ability of p300/CBP to activate NF-κB p65 by acetylation [65,66] prompted us to investigate whether NF-κB acts as a transcription factor for MCPyV gene expression.

**Fig 5:**
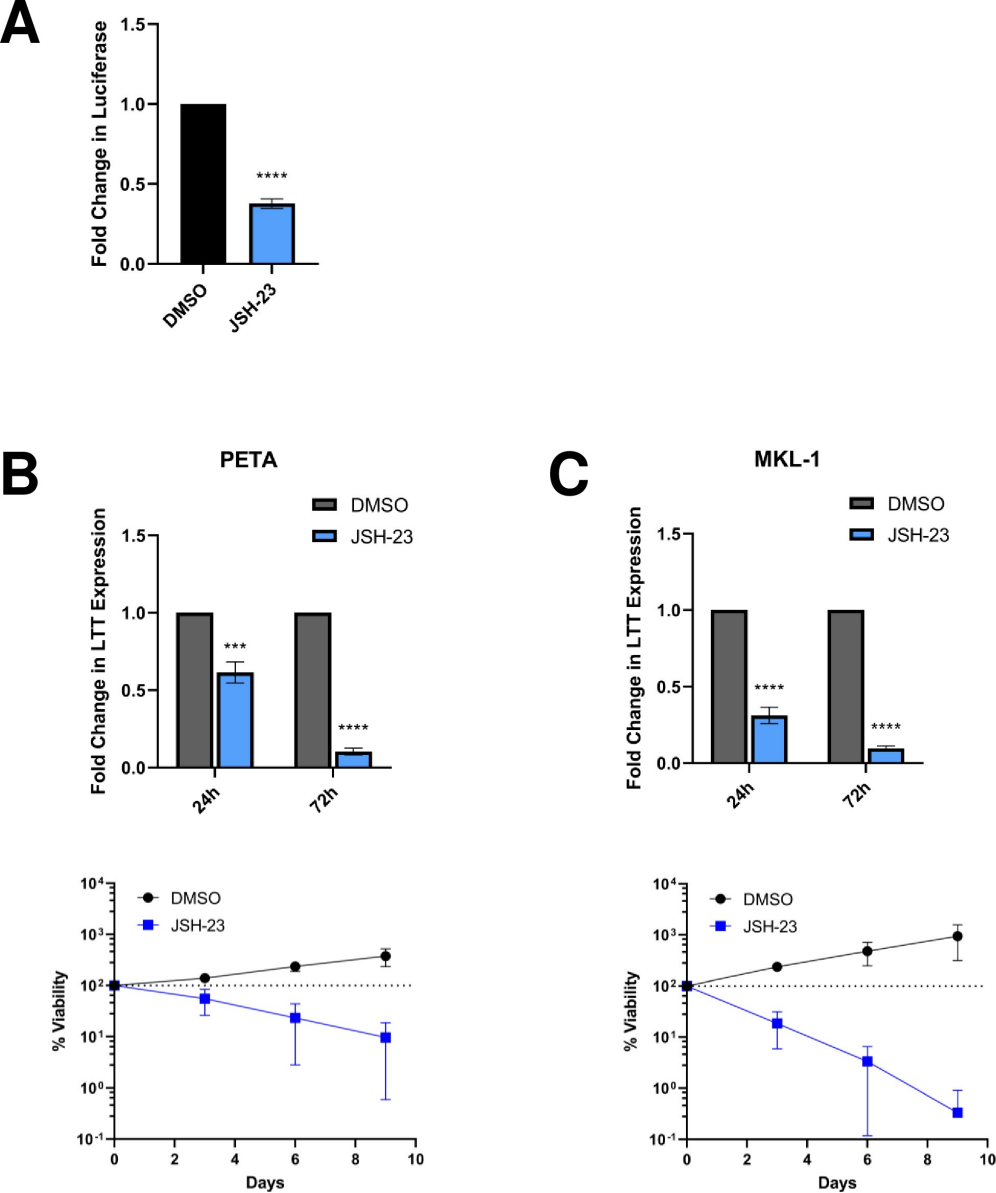
Inhibition of NF-κB activity represses MCPyV EP-driven viral oncogene expression, which is lethal in MCPyV+ MCC. (A) HEK293 cells stably expressing an MCPyV EP-luciferase reporter were treated with DMSO or 25 μM JSH-23 for 72h before EP-driven luciferase expression was measured by luciferase assay. (B) PETA and (C) MKL-1 cells were treated with DMSO or 25 μM JSH-23 for up to 9 days. At 24h and 72h post-treatment, RT-qPCR analysis was performed to measure relative changes in MCPyV LTT expression during treatment; LTT mRNA levels were normalized to the levels of cellular GAPDH mRNA. The viability of the cells was measured during treatment using the CellTiterGlo 3D assay. The % viability of the cells in each condition is expressed as the fold change in the sample’s CellTiterGlo reading relative to its d0 measurement. Error bars represent the standard deviation of three independent experiments. ****p<0.0001; ***p<0.001.

The NF-κB inhibitor (NF-κBi) JSH-23 was used to determine whether p65 activity contributes to MCPyV gene expression. NF-κB inhibition by JSH-23 reduced MCPyV EP-driven reporter expression in HEK293 cells and repressed LTT expression in the MCPyV+ MCC cell lines PETA and MKL-1 (Fig 5). In line with MCPyV+ MCCs’ addiction to constitutive viral oncoprotein expression [39], repression of LTT levels was also associated with significant cancer cell death (Fig 5B and 5C).

**Fig 6:**
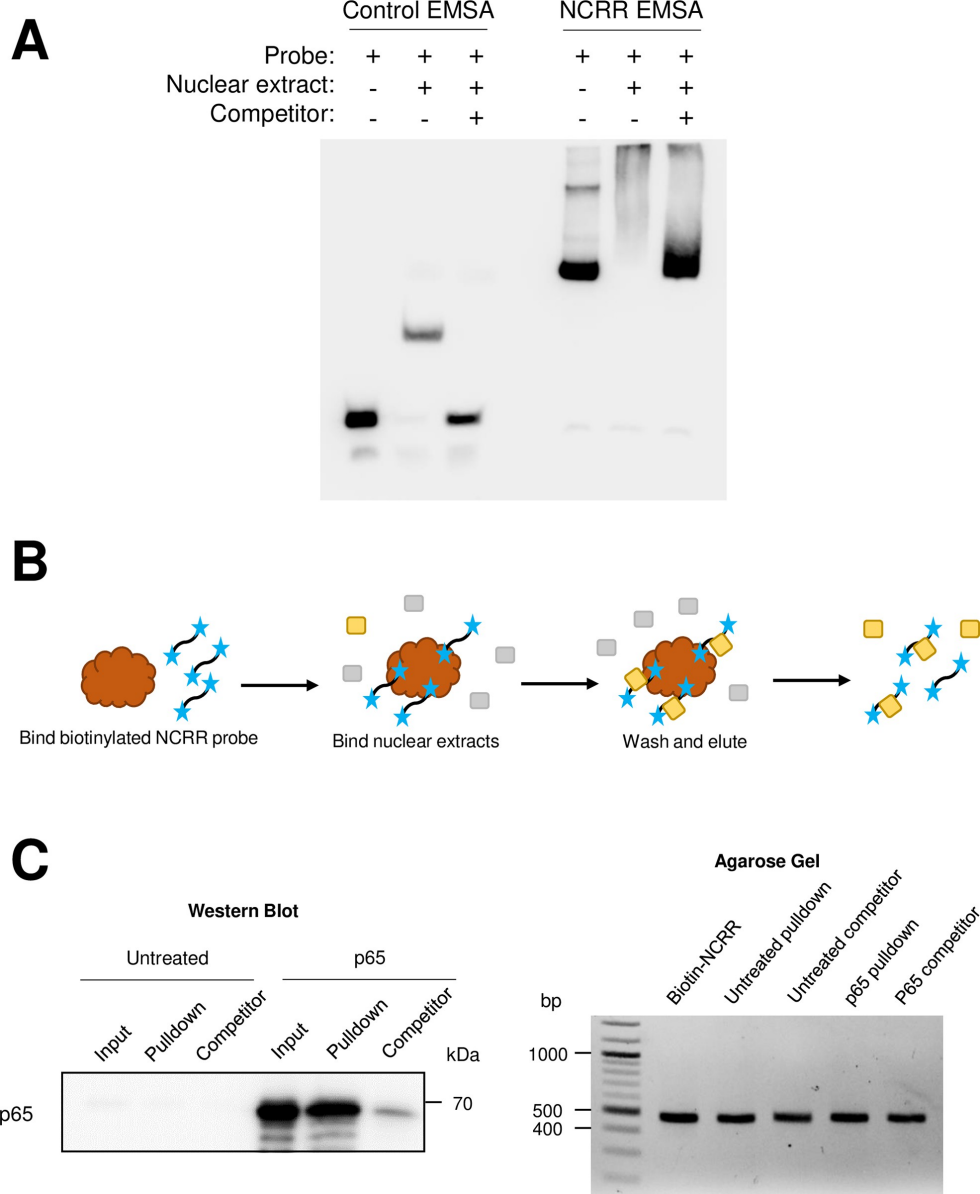
NF-κB p65 binds directly to the MCPyV NCRR. (A) NCRR-specific DNA binding activity is detected in HEK293 nuclear extracts containing overexpressed p65. EMSA was performed using a set of positive control probes and nuclear extract provided in the LightShift Chemiluminescent EMSA kit (“Control EMSA”) or using full NCRR probes and nuclear extracts from HEK293 cells transfected with a p65-expressing plasmid (“NCRR EMSA”). (B) Schematic of the biotinylated DNA pulldown assay. Biotinylated (blue stars) NCRR probes were bound to streptavidin-coated magnetic beads (brown), then incubated with nuclear extracts containing the protein of interest (in yellow; other nuclear proteins are indicated in gray). Alternatively, the nuclear extracts are pre-incubated with an excess amount of unlabeled NCRR probe before being incubated with the bead-bound probes. Protein-probe complexes (protein of interest [yellow] bound to biotinylated [blue stars] probes) are eluted off the beads for analysis by SDS-PAGE/Western blot or agarose gel electrophoresis. (C) NF-κB p65 binds the MCPyV NCRR. Biotinylated NCRR pulldown assays were performed with nuclear extracts from untreated HEK293 cells (“Untreated”), or cells overexpressing p65 (“p65”), and biotinylated-NCRR probes in the presence or absence of an excess of unlabeled NCRR competitor (“Competitor”). The left panel depicts the detection of p65 by Western blotting in the input (1%) and pulldown samples, while the right panel demonstrates that comparable amounts of biotinylated probe were bound to the beads in each pulldown experiment.

To investigate whether p65 directly binds to the MCVEP as predicted to control viral transcription, we performed an electrophoretic mobility shift assay (EMSA) to detect whether nuclear extract proteins from HEK293 cells overexpressing p65 bind to the full MCPyV NCRR (Fig 6A). We found that the labeled NCRR probe was shifted by protein present in HEK293 cells overexpressing p65 (Fig 6A). The addition of a 200-fold molar excess of unlabeled NCRR in the binding reaction as a competitor abolished the gel shift, revealing an NCRR-specific protein-DNA interaction. Due to the relatively large size of the full NCRR probe, however, we were unable to perform an antibody supershift assay to specifically detect p65 binding (Fig 6A). We therefore employed a pulldown approach to detect p65-NCRR binding. To do this, biotinylated NCRR probes were immobilized on streptavidin beads and used to pull down NCRR-binding proteins from nuclear extracts of HEK293 cells overexpressing p65, which were then detected by Western blotting (Fig 6B). Through this method, we discovered that p65 binds to the MCPyV NCRR (Fig 6C). We also found that unlabeled NCRR was able to successfully compete with the biotinylated probe for p65 binding, confirming that p65 binds the NCRR in a sequence-specific manner (Fig 6C).

**Fig 7:**
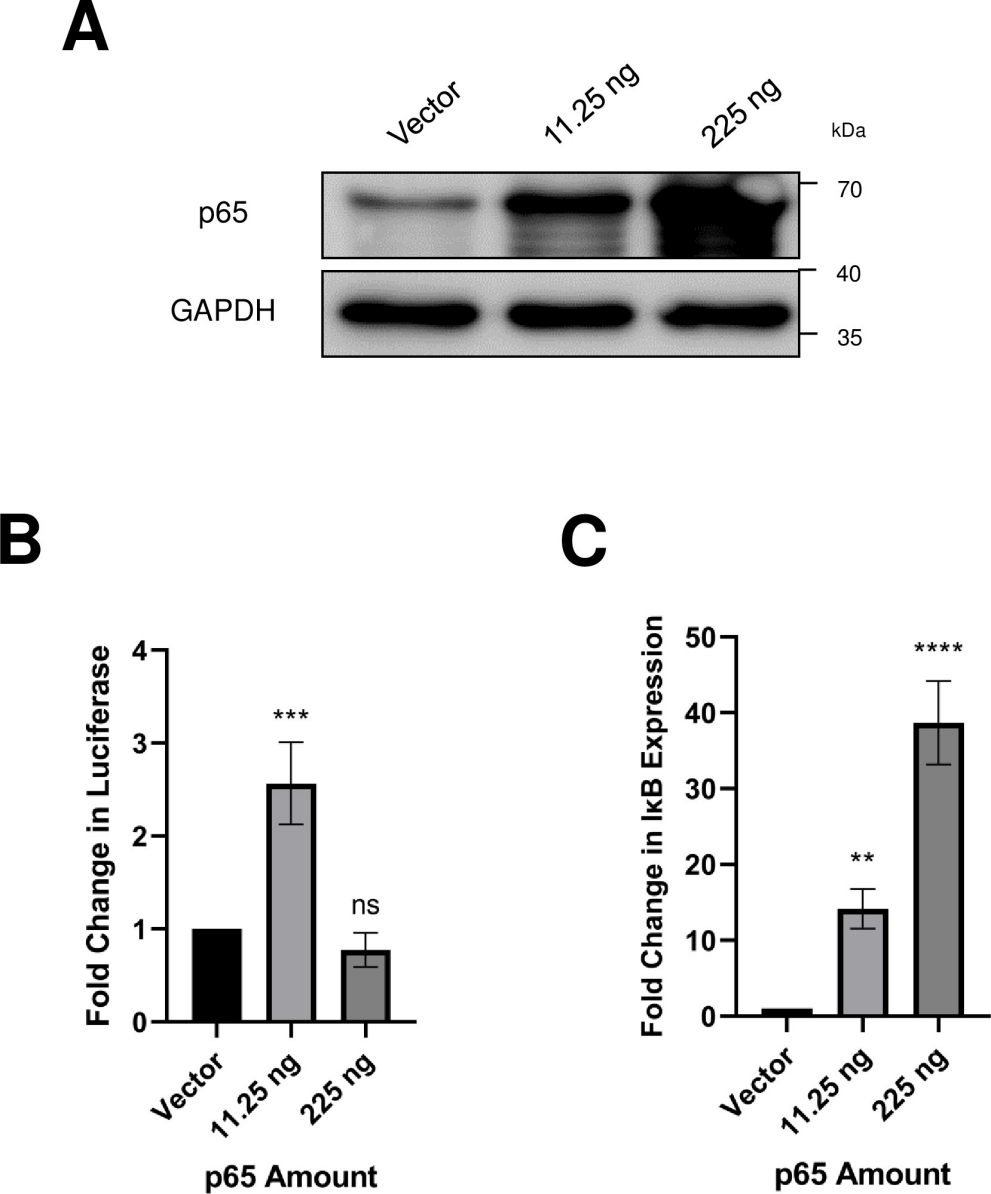
NF-κB p65 regulates MCPyV EP-driven transcription in a tightly controlled manner. HEK293 cells were transfected with an MCPyV EP-luciferase reporter, a control reporter expressing Renilla luciferase, and the indicated amounts of a p65 expression plasmid. Cells were collected 24h after transfection for Western blot analysis (A), luciferase assay (B), or RT-qPCR analysis for IκB mRNA (C). Luciferase readings were normalized to the Renilla luciferase values for each sample. Changes in IκB mRNA level were normalized to cellular GAPDH mRNA. Error bars represent the standard deviation of three independent experiments. ****p<0.0001; ***p<0.001; **p<0.01; ns = not significant.

We also sought to directly examine the positive regulation of the MCPyV EP by p65. Using an MCPyV EP-luciferase reporter, we found that low levels of exogenous p65 overexpression caused a significant upregulation in reporter expression (Fig 7A and 7B). Interestingly, higher levels of p65 overexpression caused a repression of MCPyV EP-driven reporter expression (Fig 7A and 7B). NF-κB p65 has been shown to mediate its own negative feedback loop by driving the transcription of IκBα, which acts as a multifunctional inhibitor of NF-κB by blocking its DNA binding, masking its nuclear localization signals, and mediating its export out of nucleus [67–72]. Indeed, cells transfected with a high dose of p65 plasmid showed a significant increase in IκBα expression, which may contribute to the repression of NF-κB activity on the MCPyV EP (Fig 7B and 7C). Together, these results indicate that NF-κB modulates MCPyV EP-driven transcription in a tightly regulated manner: low levels of NF-κB activate the MCPyV EP, while overstimulation of NF-κB activity results in inhibition of viral transcription, likely through the NF-κB/IκB negative feedback loop.

### NF-κB is coactivated by p300/CBP to modulate the MCPyV EP

**Fig 8:**
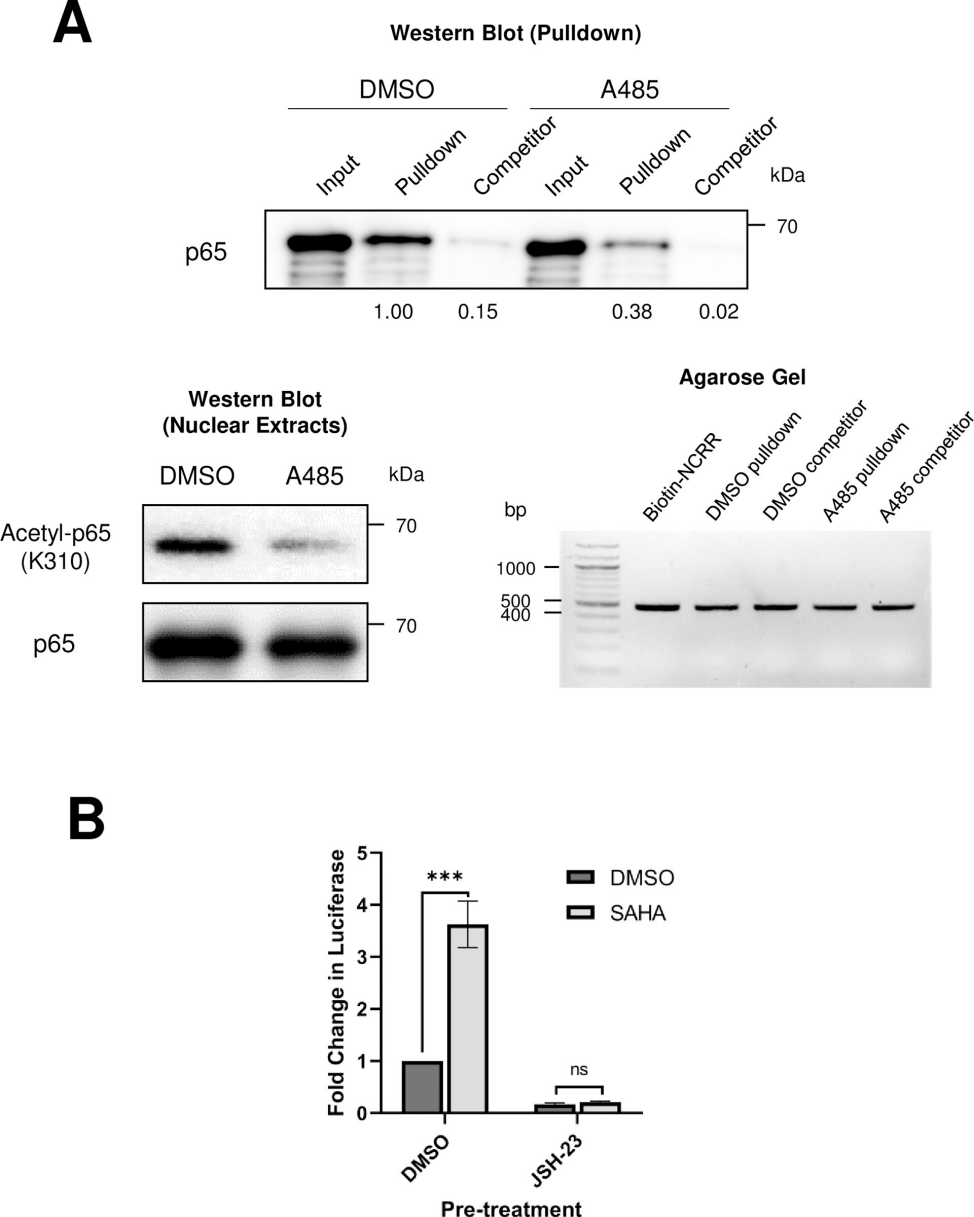
NF-κB p65 functions downstream of p300/CBP to modulate MCPyV gene expression. (A) Biotinylated NCRR pulldown assays were performed with nuclear extracts from cells pre-treated with DMSO or 2 μM A485 for 20h before transfection with a p65-expressing plasmid. Nuclear extracts were collected 24h after transfection and incubated with biotinylated-NCRR probes attached to streptavidin magnetic beads, in the presence or absence of an excess of unlabeled NCRR competitor (“Competitor”). Protein and DNA were eluted from the beads for Western blot or agarose gel analysis. The upper panel depicts the detection of p65 by Western blotting in the input (1%) and pulldown samples, with band intensities for the Pulldown and Competitor lanes relative to the DMSO Pulldown condition. The lower left panel presents the Western blotting analysis of the nuclear extracts, while the lower right panel demonstrates that comparable amounts of biotinylated probe were bound to the beads in each pulldown experiment. (B) HEK293 cells were pre-treated with DMSO or 25 μM JSH for 16h before being transfected with an MCPyV EP-luciferase reporter plasmid. 8h after transfection, cells were treated with DMSO or 1 μM SAHA, and collected for luciferase assay 20h later. Luciferase values were normalized to the total protein concentration of each sample. Error bars represent the standard deviation of three independent experiments. ***p<0.001; ns = not significant.

Having confirmed that both p300/CBP and NF-κB regulate MCPyV transcription (Figs 2, 3, 5, 6 and 7), we next investigated whether p300/CBP support MCPyV EP transcription by coactivating NF-κB to promote its DNA binding and transcriptional activity. Coactivation of NF-κB by p300/CBP requires acetylation of p65 on lysine 310, which enhances its DNA binding ability [65]. To determine whether p300/CBP acetyltransferase activity is important for NF-κB p65 to bind to the MCPyV EP, we used biotinylated MCPyV NCRR probes and tested the binding ability of p65 expressed in HEK293 cells pre-treated with the HATi A485 (Fig 8A). Similar to the results seen in Fig 6C, nuclear p65 from HEK293 cells pretreated with the DMSO vehicle control binds to the MCPyV NCRR in a sequence-specific manner (Fig 8A). In comparison, pretreating the cells with A485 to prevent p300/CBP-mediated acetylation of NF-κB caused a significant reduction in the ability of p65 to bind to the NCRR (Fig 8A). This result indicates that the HAT activity of p300/CBP is necessary for the coactivation of NF-κB and its binding to the viral genome.

We have explored two potential mechanisms through which p300/CBP regulate MCPyV gene expression: through the direct acetylation of viral genome-associated histones (Fig 4), and through the coactivation of the transcription factor NF-κB (Fig 8A). We further sought to determine the significance of NF-κB’s acetylation by p300/CBP in supporting MCPyV viral transcription. As observed previously, treatment with a low dose of HDACi upregulates expression of an MCPyV EP-luciferase reporter (S3 Fig). This treatment may stimulate MCPyV EP activity through the potentiated coactivation of NF-κB and/or the maintenance of activating histone acetylation marks on the viral EP. We reasoned that if p300/CBP primarily regulate the MCPyV EP through NF-κB-stimulating acetylation, then HDACi treatment would no longer effectively upregulate EP transcription activity if NF-κB is inhibited. To test this hypothesis, we pre-treated HEK293 cells with the NF-κBi JSH-23 prior to transfection with an MCPyV EP-luciferase reporter and HDACi treatment (Fig 8B). Cells pre-treated with a vehicle control supported increased EP-driven reporter expression in response to HDACi treatment, while the reporter no longer responded to HDACi treatment in cells that had been pre-treated with JSH-23 (Fig 8B). These results suggest that p300/CBP-mediated NF-κB acetylation is the primary mechanism through which these HATs work to regulate MCPyV EP activity.

### Blocking p300/CBP-mediated MCPyV oncogene transcription to specifically inhibit MCPyV+ MCC tumor cell growth

In MCPyV+ MCCs, the key viral oncogenes LTT and sT are transcribed from an intact MCPyV EP integrated into the MCC genome to drive tumor cell growth [11,23,29,32,33,35–42]. We therefore reason that inhibiting this promoter activity could suppress viral oncogene expression and induce a deleterious effect specifically on MCPyV+ MCCs. We have identified two key regulators of MCPyV transcription: p300/CBP, and NF-κB p65. Given their crucial roles in driving the expression of the viral oncoproteins, we decided to exploit the potential of inhibiting these factors to induce specific killing of MCPyV^+^ MCC cells in order to develop a targeted therapeutic strategy.

**Fig 9:**
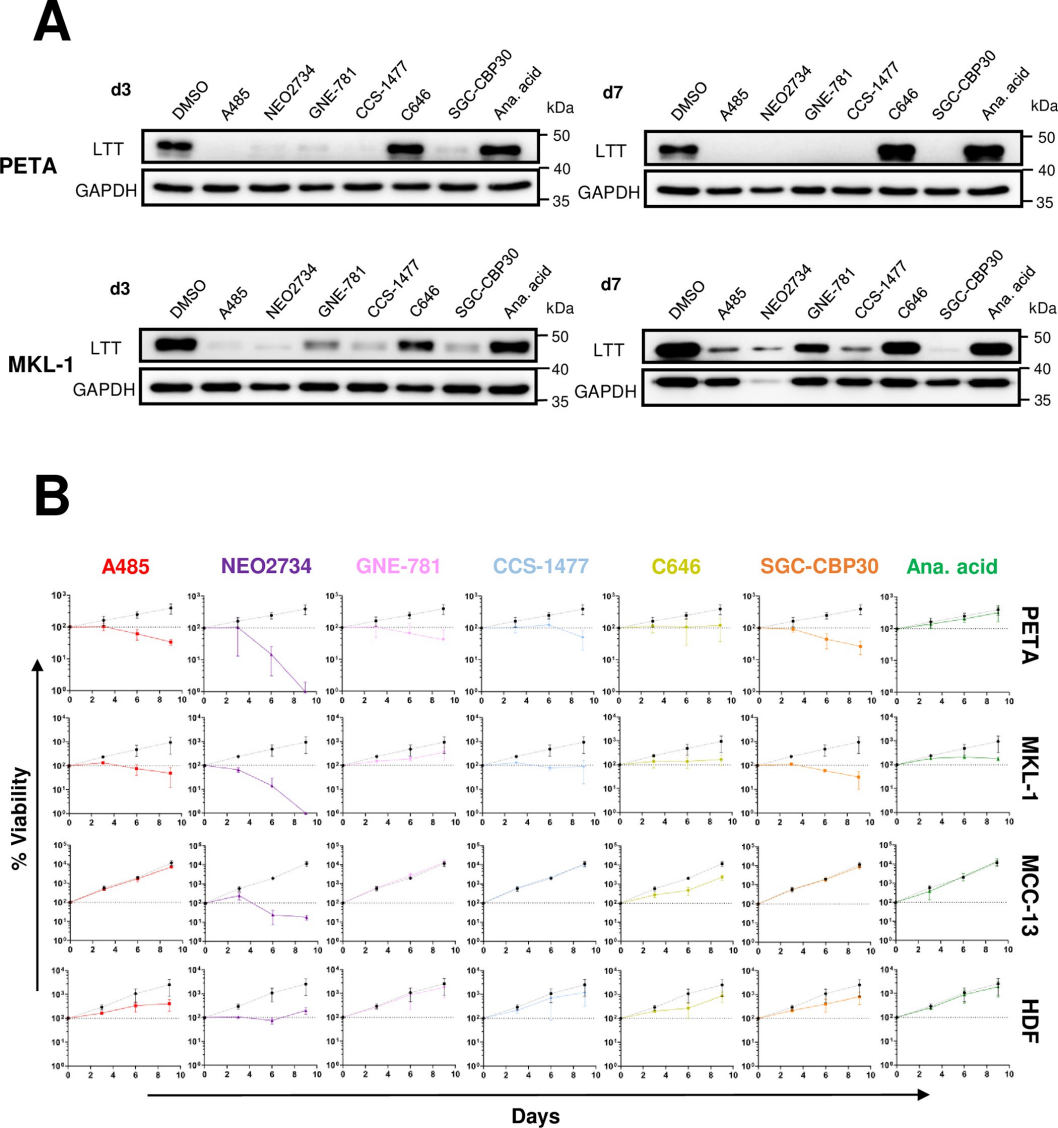
Inhibition of p300/CBP activity represses MCPyV LTT expression to specifically kill MCPyV+ MCC. (A) PETA and MKL-1 cells were treated with DMSO, 2 μM A485, 1 μM NEO2734, 1 μM GNE-781, 1 μM CCS-1477, 10 μM C646, 10 μM SGC-CBP30, or 20 μM anacardic acid. After 3 and 7 days, cell lysates were subjected to Western blotting analysis to detect MCPyV LTT and GAPDH expression. (B) PETA, MKL-1, MCC-13, and HDFs were treated with the indicated inhibitors for up to 9 days. Cell viability on days 0, 3, 6, and 9 was measured using the CellTiterGlo 3D assay. The % viability of the cells in each condition is expressed as the fold change in the sample’s CellTiterGlo reading relative to its d0 measurement. In each plot, the % viability of DMSO-treated cells is represented by a dotted black line, while the % viability of HATi-treated cells is represented by a solid colored line. Error bars represent the standard deviation of three independent experiments.

Though NF-κB inhibition by JSH-23 was effective at repressing both LTT expression and cell survival in PETA and MKL-1 (Fig 5B and 5C), it is also toxic to MCPyV- MCC cells (S4 Fig), indicating that the effects of JSH-23 treatment are not specific to the downregulation of MCPyV EP activity. We therefore focused on targeting p300/CBP to inhibit MCPyV oncogene expression in MCPyV+ MCC. To investigate whether small molecule inhibitors of p300/CBP are effective against MCPyV+ MCC growth, the cell lines PETA and MKL-1 were treated with the panel of p300/CBP-specific HATis used in the studies described above (Figs 2, S1 and S2). Generally, treatment of both PETA and MKL-1 cells with HATis caused significant repression of LTT expression (Fig 9A), coinciding with significantly reduced cell viability throughout treatment (Fig 9B). Notably, the inhibitors C646, anacardic acid, and GNE-781 (in MKL-1) were relatively ineffective at repressing LTT expression (Fig 9A); these same inhibitors were also unable to induce significant cell death in PETA and MKL-1 cells (Fig 9B). Consistently, these inhibitors were also less effective at repressing p300/CBP-specific histone H3K27 acetylation when used to treat normal HDFs (S2 Fig). This demonstrates that the effectiveness of HATi treatment against MCPyV+ MCC growth correlates with its ability to block p300/CBP acetyltransferase activity and repress the expression of the viral oncogenes.

To further verify that the ability of certain HATis to repress MCPyV+ MCC is due to the targeted downregulation of viral oncoprotein expression and not due to off-target effects, the same inhibitors were used to treat the MCPyV- MCC cell line MCC-13 (Fig 9B). Indeed, MCC-13 cells tolerated treatment by all inhibitors aside from NEO2734 (Fig 9B), which was previously found to be poorly tolerated in multiple cell types (Fig 2). Additionally, to assess the toxicity of these HATis in healthy cells, the inhibitors were used to treat healthy primary HDFs. Again, with the exception of the inhibitor NEO2734, HATi treatment was also well tolerated in HDFs (Fig 9B). Together, our studies show that HATis specific to p300/CBP are highly effective at killing MCPyV+ MCC through the repression of MCPyV transcription from the integrated viral genome.

## Discussion

**Fig 10:**
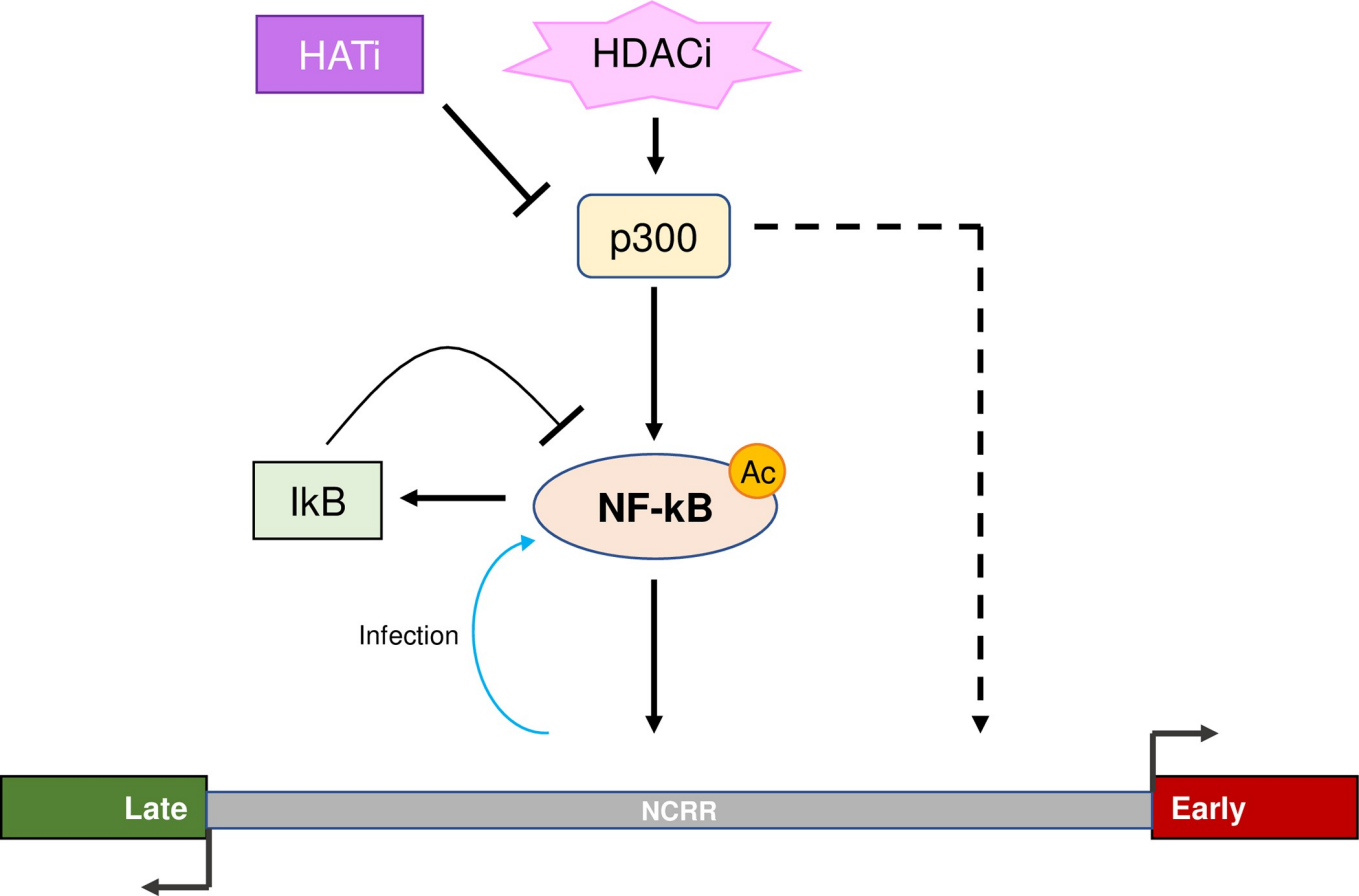
Molecular mechanisms regulating the NCRR-driven transcriptional program of MCPyV. p300/CBP upregulate MCPyV gene expression primarily through acetylation of the p65 subunit of NF-κB, which binds directly to kB site(s) on the NCRR. p300/CBP may also acetylate the histones associated with the viral NCRR to stimulate MCPyV transcription. In addition to p300/CBP-mediated acetylation, NF-κB activity is also stimulated by MCPyV infection in HDFs. Overstimulation of NF-κB induces the expression of IκBα, which in turn inhibits NF-κB activity through a negative feedback mechanism. The accumulation of NF-κB acetylation within the cell by HDAC inhibition upregulates the p300/CBP-mediated stimulation of viral transcription, while HAT inhibition robustly represses viral transcription downstream of p300/CBP.

Expression of the MCPyV tumor antigens controls many aspects of the viral life cycle, including both the establishment and maintenance of successful infection and MCPyV+ MCC oncogenesis [11,28,39]. Characterizing the molecular mechanisms regulating the expression of the viral oncogenes is therefore crucial to understanding how MCPyV infection progresses into tumorigenesis and how to target tumor antigen-addicted MCC. However, very little is known about the cellular factors that regulate MCPyV transcription during either viral infection or MCC development. In this study, we showed that the MCPyV EP regulates the cell type-specific gene expression of MCPyV (Fig 1). We then investigated which transcription factors and epigenetic enzymes contribute to MCPyV EP-mediated gene expression. Through an inhibitor screen, we identified the HATs p300 and CBP as the main epigenetic modulators of viral transcription (S1 Fig). Specific small molecule inhibitors of p300/CBP as well as siRNA-mediated knockdown of p300/CBP effectively inhibit MCPyV oncogene expression, while HDACi treatment is capable of upregulating it (Figs 2, 3 and S3). Together, we discovered that p300 and CBP positively regulate MCPyV transcription (Fig 10). These results independently corroborate those of Rapchak et al., who identified CBP as a positive regulator of MCPyV transcription [47]. Their study also showed that CBP functions as a binding partner of sT to activate MCPyV gene expression and that inhibition of CBP by A485 suppresses viral gene activation and reduces LTT expression in MCPyV+ MCC cells [47]. Though their assays did not identify p300 as an sT-interacting partner, our findings suggest that p300 likely regulates MCPyV transcription in an sT-independent manner [47].

The effectiveness of HATis at inhibiting MCPyV gene expression indicates that p300/CBP regulate viral transcription through their acetyltransferase activity. Acetylation of either DNA-binding transcription factors or viral genome-associated histones are therefore two possible mechanisms through which p300/CBP affect viral transcription [64]. We detected histone acetylation marks characteristic of p300/CBP associated with the viral EP in MCPyV+ MCC cells, confirming that the viral chromatin is directly acetylated (Figs 4 and 10). Additionally, p300/CBP are known to coactivate many transcription factors, including NF-κB, which we chose to investigate due to our previous finding that NF-κB is activated by MCPyV infection [63,64]. Results from experiments using an NF-κB specific inhibitor and exogenous expression of the p65 subunit of NF-κB indicated that NF-κB, when expressed at low levels, stimulates viral gene expression (Figs 5 and 7). We also confirmed that p65 binds directly to the viral NCRR, and that this binding is dependent on the coactivation of NF-κB by p300/CBP (Figs 6 and 8A). To investigate whether NF-κB functions downstream of p300/CBP to stimulate MCPyV transcription, we utilized a dual drug treatment approach where MCPyV EP-luciferase reporter cells were pre-treated with the NF-κBi JSH-23 prior to HDAC inhibition (Fig 8B). Treatment with only HDACi stimulates expression of the MCPyV EP-luciferase reporter (Figs S3 and 8B). However, pre-treatment with NF-κBi rendered the HDACi ineffective at upregulating EP-reporter expression, demonstrating that p300/CBP stimulate MCPyV transcription primarily by mediating NF-κB acetylation (Fig 8B). We therefore conclude that p300 and CBP interact with the MCPyV genome through a two-armed approach, in which they acetylate both the viral chromatin and the transcription factor NF-κB, which binds the viral DNA upon coactivation; however, the acetylation of NF-κB p65 appears to be the primary mechanism through which p300/CBP modulate viral gene expression (Figs 8B and 10). Together, we discovered that the transcription factor NF-κB functions downstream of p300/CBP to mediate viral transcription (Figs 5, 6, 7, 8 and 10).

Persistent MCPyV infection is maintained in a latent state, evidenced by the low level of viral DNA detectable within the skin [14] and a lack of disease state associated with infection, even in immunocompromised HIV/AIDS patients [73]. The mechanisms regulating MCPyV latency are not fully understood, but it has been shown that LT is targeted for degradation, preventing lytic infection that would be otherwise driven by robust viral replication mediated by high levels of expressed LT [18,19,26,74,75]. The restriction of LT’s expression is likely to also occur at the transcriptional level; for example, the LT proteins of polyomaviruses closely related to MCPyV are known to auto-downregulate early viral transcription by binding to the NCRR [76–78]. However, the mechanisms that repress MCPyV LT expression at the transcriptional level are largely unknown. Interestingly, we found that viral transcription is tightly controlled by NF-κB activity, such that overstimulation of NF-κB represses the MCPyV EP, potentially due to significantly increased expression of the NF-κB inhibitor IκBα (Fig 7C). Our discovery that NF-κB regulates MCPyV transcription downstream of p300/CBP suggests that upregulation of cellular acetylation levels through HDAC inhibition also stimulates NF-κB activity (Figs 8B and 10). In line with this notion, we noticed that treating MCPyV EP reporter cells with HDACis SAHA (1 μM) and TSA (150 nM) consistently stimulated reporter expression (Figs S3 and 8B). However, treatment of MCPyV-transfected cells with higher concentrations of SAHA and TSA at 2.5 uM and 300 nM respectively repressed LT expression (S1 Fig). We reasoned that, at lower concentrations, HDAC inhibition upregulates MCPyV EP reporter expression through NF-κB, while the higher concentrations used in S1 Fig may overstimulate NF-κB and cause repression of viral gene expression via the NF-κB/IκBα negative feedback loop (Figs S1, S3 and 7). The rates of viral transcription in the host cell are therefore highly sensitive to levels of NF-κB activity, such that upstream modulation of p300/CBP activity through the use of HAT or HDAC inhibitors exerts significant effects on viral gene expression (Fig 10).

Our group recently established that NF-κB is stimulated at later stages of MCPyV infection, during which NF-κB p65 is phosphorylated and translocates to the nuclei of infected cells [63]. Additionally, we discovered that this NF-κB activation also stimulates the expression of downstream proinflammatory cytokines [63,79]. Though the direct effects of these cytokines on viral activity are still unknown, interestingly, the stimulation of NF-κB during late-stage infection is associated with a peak in viral transcription, followed by a significant reduction of detectable viral transcripts in the following days [63,79]. These observations suggest that NF-κB regulates MCPyV at the transcriptional level during infection, initially by stimulating viral transcription, followed by repressing it through its negative feedback loop [63]. We have thus discovered a mechanism wherein NF-κB activated by MCPyV infection functions to tightly control viral transcription in order to maintain low-level viral gene expression and activity. This mechanism may contribute to a novel viral latency program that supports MCPyV persistent infection.

Our studies also suggest additional molecular mechanisms that may be involved in the regulation of MCPyV transcription. In our inhibitor screens, the BET inhibitor JQ1 and the dual BET/HAT inhibitor NEO2734 effectively repressed MCPyV gene expression (Figs S1, 2 and 9). The BET protein BRD4 can bind P-TEFb to stimulate transcriptional activation [80–82] and is known to interact with p300/CBP-acetylated p65 to enhance the latter’s transcriptional activity [83,84]. JQ1 functions by competitively binding the bromodomains of BET proteins, which recognize acetylated lysine residues such as those present on histones or transcription factors [52,85]. We also previously discovered that BRD4 localizes to MCPyV genome in the infected cells [86]. We therefore hypothesize that BRD4 interacts with acetylated p65 or acetylated viral chromatin to stimulate the transcriptional activity of MCPyV EP.

During this study, we sought to determine whether the mechanisms modulating MCPyV oncoprotein expression could be targeted to treat MCPyV+ MCC. Though NF-κB inhibition repressed the growth of MCPyV+ MCC cells, the inhibitor used in these experiments was also toxic to other cell types, including MCPyV- MCC, making it unsuitable as a treatment strategy (Figs 5 and S4). Certain HATis, however, were highly effective at reducing LTT expression and suppressing the viability of MCPyV+ MCC, yet were still well-tolerated in MCPyV- MCC and healthy HDFs (Fig 9). The HATis C646 and anacardic acid, which were toxic in several cell types yet ineffective at repressing LTT expression and MCPyV+ MCC survival, also poorly repressed the p300/CBP-specific H3K27 acetylation in HDFs, indicating that potent inhibition of p300/CBP activity is crucial to targeting MCPyV+ MCC (S2 Fig). Our finding that p300/CBP-specific HATis cause significant killing of MCPyV+ MCC cells is exciting because, in the *in vivo* setting, tumor antigens released by dying MCC cells could be engulfed by antigen-presenting cells (APCs) to activate T cells, which can then kill more tumor cells and amplify the tumoricidal effect [87]. Together, the data presented in this study therefore support the use of p300/CBP-specific HATis as a potential targeted treatment strategy against MCPyV+ MCC. Though several of the inhibitors used in this study, such as NEO2734, C646, and anacardic acid, are unsuitable for therapeutic use due to their toxicity or lack of selectivity for p300/CBP (Figs S2, 2 and 9) [55,60,61], others may be suitable for future patient use. Among these HATis, A485 and CCS-1477 hold the greatest promise for future preclinical studies, owing to their high specificity against p300/CBP, potent and orally bioavailable formulation, relatively robust efficacy at HAT inhibition, and specific toxicity in only MCPyV+ MCC (Figs S2 and 9) [53,56,88]. Furthermore, CCS-1477 is currently in phase I/IIA clinical trials for multiple other cancer types [89–91], while A485 has been well-tolerated and effective in various *in vivo* studies [92–94], supporting their viability as effective and safe drug candidates against MCPyV+ MCC. Additionally, the HATi SGC-CBP30 was also highly effective in our experiments (Figs S2 and 9) and has been successfully used in *in vivo* studies despite prior observations that the compound is metabolized too rapidly for use as an oral drug [95,96]. SGC-CBP30 may therefore also have potential as a treatment against MCPyV+ MCC.

In summary, this study represents the first attempt to characterize the molecular mechanisms regulating MCPyV transcription. We discovered that p300 and CBP upregulate MCPyV gene expression through coactivation of NF-κB p65, which binds directly to the viral EP as a transcription factor, and potentially through the direct acetylation of the viral chromatin (Figs 4, 8 and 10). Furthermore, this mechanism can be exploited to kill MCPyV+ MCC through the specific downregulation of viral transcription by small molecule inhibitor treatment (Figs 9 and 10). Through this work, we demonstrate that targeting MCPyV gene expression is a novel, effective, and highly specific approach for treating MCPyV+ MCC. This treatment strategy will be improved with future discoveries of the additional factors regulating MCPyV transcription, which will provide the basis for new targeted treatments against this aggressive cancer.

## Materials and methods

### Cell culture and reagents

The protocol for the isolation of primary HDFs has been described previously [31]. Primary HDFs, HEK293, HEK293T, C33A, HeLa, and HaCaT cells were maintained in Dulbecco’s modified Eagle medium (DMEM) (Life technologies) supplemented with 10% FBS (HyClone), 1x nonessential amino acids (Gibco), and 1x glutamine (Gibco). MKL-1, PETA, and MCC-13 cells were maintained in RPMI 1640 medium (Gibco) supplemented with 20% FBS.

Inhibitors used in this study are listed in the Table 1 below. BIX01294 was reconstituted in H_2_O, while all others were reconstituted in DMSO. Aliquots of inhibitor stocks were stored at -80°C.

**Table 1:** Chemical inhibitors used in this study.

Inhibitor	Catalogue Number
JQ1	ApexBio A1910
5-AZADC	Sigma A3656
Zebularine	Sigma Z4775
BIX01294	Sigma B9311
UNC0642	Sigma SML1037
GSK126	Thomas Scientific C818K14
UNC1999	Sigma SML0778
A196	Sigma SML1565
A485	Sigma SML2192
NEO2734	Selleckchem S9648
CCS-1477	Selleckchem S9667
GNE-781	Selleckchem S8665
C646	Sigma SML0002
SGC-CBP30	Sigma SML1133
Anacardic acid	Sigma A7236
SAHA	Sigma SML0061
Trichostatin A	Sigma T8552
Belinostat	ApexBio A4096
Panobinostat	ApexBio A8178
Romidepsin	ApexBio A8173
JSH-23	Sigma J4455

The following inhibitor concentrations were selected based on their use in published studies (listed below), in which the selected concentrations were found to be effective in various cell types: 2 μM A485 [97,98], 1 μM NEO2734 [55], 1 μM GNE-781 [54], 1 μM CCS-1477 [53], 10 μM C646 [58,99], 10 μM SGC-CBP30 [57,100], and 20 μM anacardic acid [59,101].

### Recombinant plasmid construction

*pLenti-MCPyVEP-tRFP-UBC-Puro*: The MCPyV early promoter (MCPyV EP) was PCR-amplified from the pR17b plasmid (kindly provided by Dr. Christopher B. Buck, NCI) using primers described previously [102]. The resulting fragment was subcloned using XbaI and AgeI sites into the pTRIPZ vector (Open Biosystems).

*pLenti-HPV11 LCR-tRFP-UBC-Puro*: The HPV11 LCR was PCR-amplified from the HPV11 genome using the primers listed below. The resulting fragment was subcloned using the XbaI and AgeI sites into the pTRIPZ vector (Open Biosystems).

HPV11LCR F: GCTCTAGAGGATCCCTATAAGGATATGAGTTTTTGG

HPV11LCR R: CCCCCCGGGAATGCCTCGTCTGCTAATTTTTTGG

*pLenti-MCPyVEP-Luciferase-IRES-Puro*: The UBC promoter and rtTA3 element were removed from the pTRIPZ vector (Open Biosystems) using BamHI. The MCPyV EP was PCR-amplified from the pR17b plasmid and subcloned into the vector using the XbaI and AgeI sites. The firefly luciferase gene was PCR-amplified from the pGL3 Basic reporter (Promega) using the primers listed below. The resulting fragment was subcloned into the vector using the AgeI and ClaI sites.

Luciferase (AgeI) F: GCGACCGGTCGCCACCATGGAAGACGCCAAAAACATAAAG

Luciferase (ClaI) R: CCATCGATTACACGGCGATCTTTCCGCC

MCPyV genomes were digested out of pR17b using BamHI sites and religated. The T7-RelA (p65) plasmid was a gift from Warner Greene (Addgene 21984). pRL-SV40 (Renilla luciferase reporter) was purchased from Promega (E2231).

### Transfection and lentiviral transduction

Lipofectamine 2000 (Invitrogen) was used for transient transfection of MCPyV genomes, pLenti-MCPyVEP-Luciferase-IRES-Puro, pRL-SV40, and T7-RelA according to the manufacturer’s instructions.

To generate HDF, MKL-1 and HaCaT cells stably expressing MCPyVEP-tRFP and HPV11 LCR-tRFP, pLenti-MCPyVEP-tRFP-UBC-Puro or pLenti-HPV11 LCR-tRFP-UBC-Puro were transfected into HEK293T cells together with psPAX2 and pMD.2G using Lipofectamine 2000 (Invitrogen). The medium on the HEK293T cells was replaced with fresh medium at 8 hours post-transfection. Twenty-four hours later, lentiviruses were harvested from the supernatant and filtered through a 0.45μm filter. Purified lentiviruses supplemented with polybrene were used to infect HDFs, MKL-1, or HaCaT cells. HaCaT cells were treated with 5 μg/ml puromycin for two weeks, HDF cells were treated with 2 μg/ml puromycin for two weeks, and MKL-1 cells were treated with 1 μg/ml puromycin for two and a half weeks to select for transduced cells.

To generate HDF and HEK293 cells stably expressing MCPyVEP-Luciferase, pLenti-MCPyVEP-Luciferase-IRES-Puro was used to generate lentivirus as described above. Transduced HDFs and HEK293 cells were treated with 2 μg/ml puromycin for 2 weeks to select for stable cells.

### siRNA knockdown

SMARTPool EP300 siGENOME siRNA (M-003486-04-0010) and SMARTPool CREBBP ON-TARGETplus siRNA (L-003477-00-0010) were purchased from Horizon Discovery/Dharmacon. siRNA transfection in HDFs were performed using DharmaFECT 3 (Horizon Discovery/Dharmacon) according to the manufacturer’s instructions.

### MCPyV infection

MCPyV virions were prepared and used to infect HDFs as described previously [31]. 10^4^ HDFs were seeded in each well of a 96-well plate and treated with 1-2x10^8^ genome equivalents of MCPyV. For experiments with p300/CBP KD HDFs, cells were seeded for infection 24 hours after the siRNA transfection. Infected cells were collected on days 3 through 6 post-infection for RT-qPCR analysis. For HATi experiments, the indicated inhibitors were added to the cells on day 2 post-infection, and cells were harvested on day 5 post-infection for RT-qPCR or CellTiterGlo 3D analysis.

### Immunofluorescent staining

Immunofluorescent staining of MCPyV-transfected C33A and HeLa cells was performed as described previously [63]. Cells were stained with antibody against the MCPyV LT (Santa Cruz sc-136172, 1:500) and counterstained with DAPI.

### Western blot analysis

To obtain whole cell lysates, cells were lysed in buffer containing 10 mM HEPES (pH 7.9), 500 mM NaCl, 3 mM MgCl_2_, 1 mM dithiothreitol (DTT), and 0.5% Triton X-100 supplemented with protease inhibitors. To obtain histone extracts for examination of histone acetylation levels, cells were washed in ice-cold PBS supplemented with 10 mM sodium butyrate, then lysed in PBS containing 0.5% Triton X-100, 2 mM phenylmethylsulfonyl fluoride (PMSF), 0.02% NaN3, and 5 mM sodium butyrate. The nuclei were pelleted and washed in lysis buffer before resuspension in 0.2 N HCl overnight to extract the histones. The nuclear debris was then pelleted before the supernatant (containing histones) was neutralized using 2M NaOH.

The protein concentrations of all samples were measured using the Bradford assay. Equal amounts of protein in each sample were resolved on SDS-PAGE gels. The protein samples in the resolved gel were either stained with the Colloidal Blue Staining Kit (Invitrogen) according to the manufacturer’s instructions or transferred to PVDF membranes and immunoblotted with the following primary antibodies: anti-H3K27ac (Cell Signaling Technology 8172, 1:1000), anti-p300 (Sigma 05–257, 1:1000), anti-CBP (Cell Signaling Technology 7389, 1:1000), anti-p65 (Cell Signaling Technology 8242, 1:1000), anti-MCPyV LT (Santa Cruz sc-136172, 1:1000), and anti-GAPDH (Cell Signaling Technology 5174, 1:5000). HRP-linked anti-rabbit IgG (Cell Signaling Technology 7074, 1:3000) and HRP-linked anti-mouse IgG (Cell Signaling Technology 7076, 1:3000) were used as secondary antibodies. The blots were developed using SuperSignal West Pico PLUS Chemiluminescent Substrate (Thermo Fisher), and images were captured on an Amersham Imager 600 (GE Healthcare/Cytiva).

### Reverse transcription (RT) and quantitative real-time PCR (qPCR)

Total RNA was isolated from cells using the NucleoSpin RNA XS Kit (Macherey-Nagel). Reverse transcription (RT) of total RNA was performed in a 20 μL reaction mixture containing 350 ng of RNA, a 1:1 mix of random hexamer primers (Invitrogen) and Oligo(dT) 12–18 primers (Invitrogen), dNTPs (Invitrogen), and M-MLV reverse transcriptase (Invitrogen). qPCR was performed using the PowerUp SYBR Green Master Mix (Applied Biosystems) and analyzed on a QuantStudio 3 Real-Time PCR System (Applied Biosystems). The mRNA levels of each gene were normalized to the mRNA levels of GAPDH or beta-actin, as indicated. Sequences of the primers used are listed in Table 2. All primers were synthesized by Integrated DNA Technologies (IDT).

**Table 2:** Sequences of the primers used in this study.

Target	Forward Primer	Reverse Primer
MCPyV LT	TGACTTCTCTATGTTTGATGAGGTTGAC	GACCCATACCCAGAGGAAGAG
MCPyV VP1	GCTTGTTAAAGGAGGAGTGG	GATCTGGAGATGATCCCTTTG
IκB	CCTGGACTCCATGAAAGACG	GGGGGTATTTCCTCGAAAGTC
GAPDH	GGTGGTCTCCTCTGACTTCAACA	GTTGCTGTAGCCAAATTCGTTGT
Beta actin	TGATGATATCGCCGCGCTCGTCGT	CACAGCCTGGATAGCAACGTACAT

### Luciferase and cell viability assays

Luciferase assays were performed with the Luciferase Assay System (Promega) or the Dual-Luciferase Reporter Assay System (Promega) according to the manufacturer’s protocol. For cell viability assays, the CellTiterGlo 3D Cell Viability Assay (Promega) was used according to the manufacturer’s protocol.

### Chromatin immunoprecipitation (ChIP)

ChIP-qPCR was performed as previously described [103]. Chromatin from 1E07 MKL-1 cells (untreated, or treated for 1 hour with the indicated inhibitors) was pulled down with 0.5 μg of normal rabbit IgG (Cell Signaling Technology 2729) or antibody against H3K27ac (Cell Signaling Technology 8173). The following primers were used for qPCR:

MCPyV EP F: GGCAGTATCTAAGGGCAG

MCPyV EP R: GACTAAATCCATCTTGTCTATATGC

GAPDH promoter F: GCTCCAATTCCCCATCTCAG

GAPDH promoter R: GCAGCAGGACACTAGGGAGT

### Electrophoretic mobility shift assay (EMSA)

The full MCPyV NCRR was PCR-amplified from pR17b using the primers listed below. The Pierce Biotin 3’ End DNA Labeling Kit (Thermo Scientific 89818) was used to label the ends of the double-stranded NCRR according to the manufacturer’s instructions.

MCPyV NCRR full F: CCCCATCCTGAAAAATAAATAAG

MCPyV NCRR full R: GACTAAATCCATCTTGTCTATATGC

EMSA assays were performed using the LightShift Chemiluminescent EMSA Kit (Thermo Scientific 20148) per the manufacturer’s protocol. Nuclear extracts from HEK293 cells transfected with a p65-expressing plasmid were used in binding reactions. Each binding reaction contained 1x binding buffer, 50 ng/μl poly(dI-dC), 2.5% glycerol, 5 mM MgCl2, 0.05% NP-40, 10 μg of nuclear extracts, and 20 fmol of biotin end-labeled NCRR probe. For competitor conditions, 4 pmol of unlabeled probe was also included in the reaction. Binding reactions were carried out for 20 minutes at room temperature before being resolved on 5% polyacrylamide/0.5x TBE gels. DNA-protein complexes were transferred to a positively-charged nylon membrane (Amersham Hybond-N+, GE Healthcare/Cytiva RPN303B), UV-crosslinked, and detected according to the kit protocol. Images were captured on an Amersham Imager 600.

### Biotinylated DNA pulldown assay

Biotinylated (5’) full MCPyV NCRR was PCR-amplified from pR17b using primers synthesized by IDT, listed below.

MCPyV NCRR full F 5’biotin: /5Biosg/CCCCATCCTGAAAAATAAATAAG

MCPyV NCRR full R 5’biotin: /5Biosg/GACTAAATCCATCTTGTCTATATGC

For each pulldown reaction, 10 μl of streptavidin magnetic beads (New England Biolabs S1420S) were incubated with 6 μg of biotinylated NCRR probe in binding buffer (10 mM Tris pH 7.5, 50 mM KCl, 1 mM DTT, 2.5% glycerol, 5 mM MgCl_2_, 10 mM EDTA supplemented with protease and HDAC inhibitors) for 20 minutes at 4°C, and then washed in fresh binding buffer. The probe-coated beads were incubated with 300 μg of HEK293 nuclear extracts in binding buffer supplemented with 50 ng/μl poly[d(I-C)] for 1.5 hours at room temperature. For competitor conditions, the nuclear extracts were pre-incubated with 160 μg of unlabeled NCRR probe in binding buffer supplemented with poly[d(I-C)] for 20 minutes at 4°C prior to adding them to the beads. After protein-bead incubation, the beads were washed 3x with binding buffer. Each sample was then divided in half. One half of each sample was eluted with SDS sample buffer for SDS-PAGE and Western blot analysis. For analysis of the bead-bound probes, the other half of the sample was vortexed in phenol:chloroform:isoamyl alcohol (25:24:1) to elute and purify DNA. The aqueous phase was further purified with chloroform, and the DNA was ethanol precipitated and resuspended in Tris-EDTA buffer for agarose gel electrophoresis on a 2% gel.

### Statistical analysis

Statistical analysis was performed using the unpaired t-test of GraphPad Prism software (Version 9.5) to compare results between the control and experimental groups. A two-tailed P value of <0.05 was considered statistically significant.

## Supporting information

S1 Fig Inhibition of histone acetyltransferases represses MCPyV LT expression. (A) Inhibitors of different classes of epigenetic enzymes. (B) HeLa and C33A cells were transfected with religated MCPyV genomes at 5h before treatment with the inhibitors indicated in (A): 5 μM 5-AZADC, 250 μM zebularine, 4.5 μM BIX01294, 1 μM UNC0642, 5 μM GSK126, 2 μM UNC1999, 1 μM A196, 30 μM anacardic acid, 20 μM C646, 2 μM SGC-CBP30, 2 μM A485, 1 μM JQ1, 2.5 μM SAHA, and 300 nM TSA. At 16h after inhibitor treatment, cells were subject to IF analysis. LT+ cells in IF images were quantified, and changes in LT expression are represented as the fold change in % LT+ cells in inhibitor-treated cells over vehicle-treated cells. Error bars represent the standard deviation of three independent experiments. ****p<0.0001; ***p<0.001; **p<0.01; ns = not significant.
(PDF)

S2 Fig Validating the efficacy of p300/CBP-specific HATis. HDFs were treated with DMSO, 2 μM A485, 1 μM NEO2734, 1 μM GNE-781, 1 μM CCS-1477, 10 μM C646, 10 μM SGC-CBP30, or 20 μM anacardic acid for 72h before the histones were extracted and subject to SDS-PAGE, followed by either Coomassie staining to assess total histone levels or Western blot analysis to detect the p300/CBP-specific histone acetylation mark H3K27ac.
(PDF)

S3 Fig HDACi treatment upregulates MCPyV EP-driven transcription. HEK293 cells were transfected with pTRIPZ MCPyV EP-luciferase, and then treated with DMSO, 1 μM SAHA, 100 nM TSA, 1 μM Belinostat, 100 nM Panobinostat, or 250 nM Romidepsin at 8h post-transfection. The cells were collected for luciferase assay 16h after inhibitor treatment. Luciferase readings were normalized to the total protein concentration of each sample. Error bars represent the standard deviation of three independent experiments. **p<0.01; *p<0.05; ns = not significant.
(PDF)

S4 Fig NF-κB inhibition also suppresses the growth of MCPyV- MCC. MCC-13 cells were treated with DMSO or 25 μMJSH-23 for up to 9 days. Cell viability during treatment was measured using the CellTiterGlo 3D assay. The % viability of the cells in each condition is expressed as the fold change in the sample’s CellTiterGlo reading relative to its d0 measurement. Error bars represent the standard deviation of three independent experiments.
(PDF)

S1 Data Numerical data and statistical significance values for Figs 2C, 3B, 4A, 4B, 5A, 5B, 5C, 7B, 7C, 8B and 9B. (XLSX)
