# Supplementary material for: Characterization of molecular mechanisms driving Merkel cell polyomavirus oncogene transcription and tumorigenic potential
Source: PLoS Pathog. 2023 Aug 30;19(8):e1011598. doi: 10.1371/journal.ppat.1011598 (PMC10468096; doi:10.1371/journal.ppat.1011598)

S1 Fig

A

Inhibitor	Target
C646	p300 histone acetyltransferase
Anacardic acid	Histone acetyltransferases (p300, PCAF, Tip60, etc)
SGC-CBP30	p300 histone acetyltransferase
A485	p300 histone acetyltransferase
JQ1	BET proteins
5-AZADC	DNA methyltransferases
Zebularine	DNA methyltransferases
SAHA	Histone deacetylases
Trichostatin A	Histone deacetylases
BIX01294	G9a/GLP (H3K9me1/2) histone methyltransferases
UNC0642	G9a/GLP (H3K9me1/2) histone methyltransferases
GSK126	EZH2 (H3K27me3) histone methyltransferase
UNC1999	EZH2 (H3K27me3) histone methyltransferase
A196	Suv420h1/2 (H4K20me2/3) histone methyltransferases

B

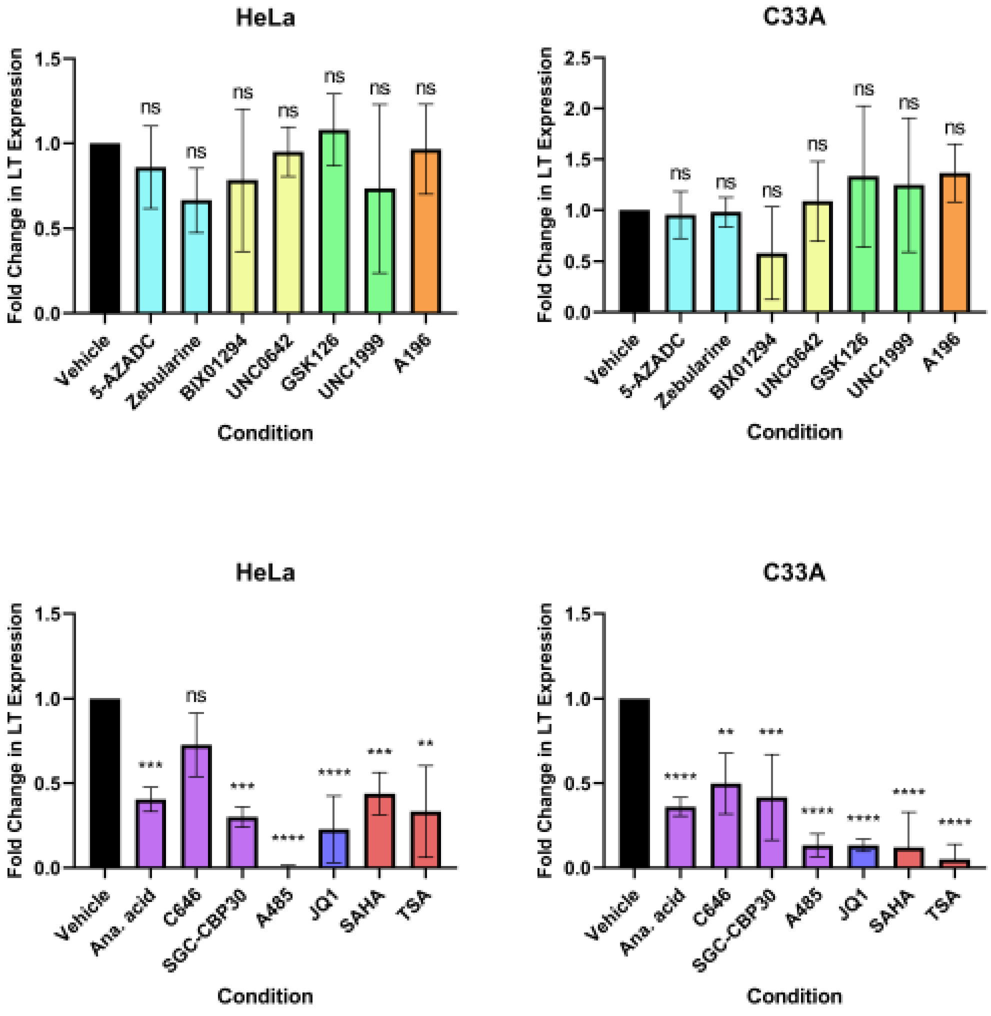

Supplement: S1 Fig — (A) Inhibitors of different classes of epigenetic enzymes. (B) HeLa and C33A cells were transfected with religated MCPyV genomes at 5h before treatment with the inhibitors indicated in (A): 5 μM 5-AZADC, 250 μM zebularine, 4.5 μM BIX01294, 1 μM UNC0642, 5 μM GSK126, 2 μM UNC1999, 1 μM A196, 30 μM anacardic acid, 20 μM C646, 2 μM SGC-CBP30, 2 μM A485, 1 μM JQ1, 2.5 μM SAHA, and 300 nM TSA. At 16h after inhibitor treatment, cells were subject to IF analysis. LT+ cells in IF images were quantified, and changes in LT expression are represented as the fold change in % LT+ cells in inhibitor-treated cells over vehicle-treated cells. Error bars represent the standard deviation of three independent experiments. ****p<0.0001; ***p<0.001; **p<0.01; ns = not significant. (PDF) [file ppat.1011598.s001.pdf]
