# Supplementary figures and images for: Characterization of molecular mechanisms driving Merkel cell polyomavirus oncogene transcription and tumorigenic potential
Source: PLoS Pathog. 2023 Aug 30;19(8):e1011598. doi: 10.1371/journal.ppat.1011598 (PMC10468096; doi:10.1371/journal.ppat.1011598)

S2 Figure

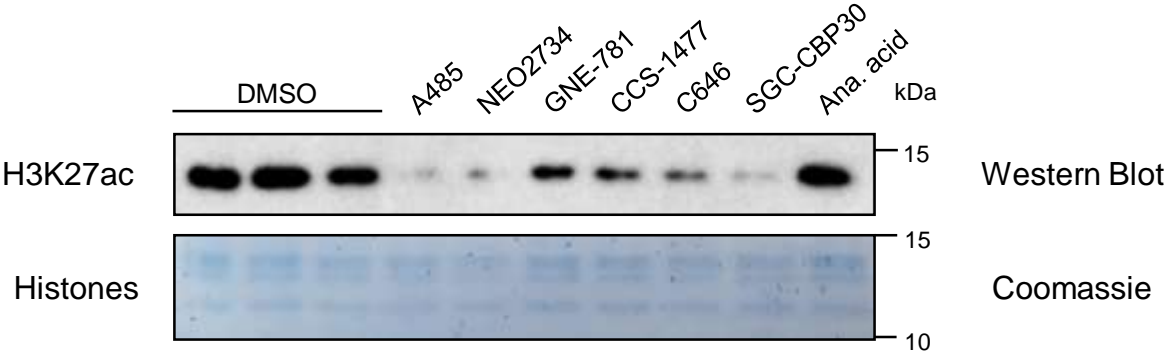

Supplement: S2 Fig — HDFs were treated with DMSO, 2 μM A485, 1 μM NEO2734, 1 μM GNE-781, 1 μM CCS-1477, 10 μM C646, 10 μM SGC-CBP30, or 20 μM anacardic acid for 72h before the histones were extracted and subject to SDS-PAGE, followed by either Coomassie staining to assess total histone levels or Western blot analysis to detect the p300/CBP-specific histone acetylation mark H3K27ac. (PDF) [file ppat.1011598.s002.pdf]

S3 Figure

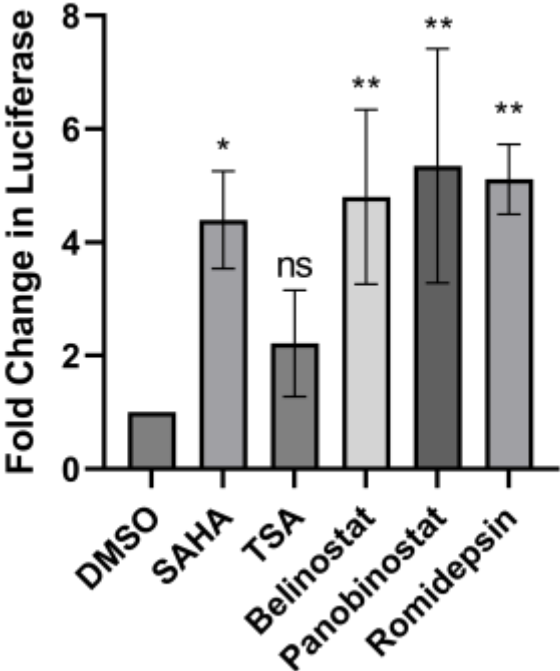

Supplement: S3 Fig — HEK293 cells were transfected with pTRIPZ MCPyV EP-luciferase, and then treated with DMSO, 1 μM SAHA, 100 nM TSA, 1 μM Belinostat, 100 nM Panobinostat, or 250 nM Romidepsin at 8h post-transfection. The cells were collected for luciferase assay 16h after inhibitor treatment. Luciferase readings were normalized to the total protein concentration of each sample. Error bars represent the standard deviation of three independent experiments. **p<0.01; *p<0.05; ns = not significant. (PDF) [file ppat.1011598.s003.pdf]

S4 Figure

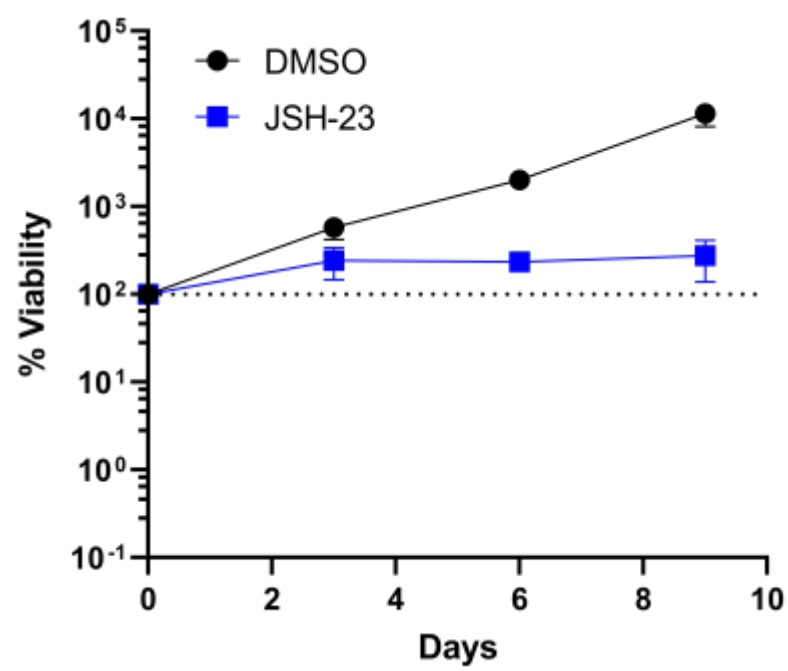

Supplement: S4 Fig — MCC-13 cells were treated with DMSO or 25 μMJSH-23 for up to 9 days. Cell viability during treatment was measured using the CellTiterGlo 3D assay. The % viability of the cells in each condition is expressed as the fold change in the sample’s CellTiterGlo reading relative to its d0 measurement. Error bars represent the standard deviation of three independent experiments. (PDF) [file ppat.1011598.s004.pdf]
